# LRRK2 I1371V Mutation Drives Astrocytic Glucose Metabolism Failure and Induces Integrated ER–Mitochondria–Lysosome Dysfunction in Parkinson’s Disease

**DOI:** 10.3390/ijms27083463

**Published:** 2026-04-12

**Authors:** Roon Banerjee, Rashmi Santhoshkumar, Vikram Holla, Nitish Kamble, Ravi Yadav, Pramod Kumar Pal, Indrani Datta

**Affiliations:** 1 Department of Biophysics, National Institute of Mental Health and Neurosciences, Institute of National Importance, Bengaluru 560029, Karnataka, India; 2 Department of Neuropathology, National Institute of Mental Health and Neurosciences, Institute of National Importance, Bengaluru 560029, Karnataka, India; 3 Department of Neurology, National Institute of Mental Health and Neurosciences, Institute of National Importance, Bengaluru 560029, Karnataka, India

**Keywords:** glial metabolic vulnerability, LRRK2 GTPase domain mutation, astrocyte metabolism, Parkinson’s disease, ER–mitochondria–Lysosome dysfunction

## Abstract

Although LRRK2 mutations modulate systemic glucose homeostasis and metabolic dysfunction precedes Parkinson’s disease (PD) motor symptoms; the way in which pathogenic variants of LRRK2 disrupt astrocytic glucose metabolism and organellar homeostasis remains poorly understood. Here, we demonstrate that LRRK2-I1371V mutation causes profound metabolic and organellar dysfunction in LRRK2-I1371V PD-iPSC-derived astrocytes and U87 cells overexpressing I1371V variant. LRRK2-I1371V astrocytes exhibit significantly reduced GLUT1 expression and cell surface localization, resulting in impaired glucose uptake and decreased lactate production. This metabolic insufficiency correlates with cascading mitochondrial dysfunction, characterized by membrane depolarization, elevated reactive oxygen species, enhanced ubiquitination and reduced proteasomal activity. Reduced LAMP1/LAMP2 expression, impaired lysosomal acidification, and selective cathepsin D deficiency were observed. Accumulation of undegraded cargo was confirmed by transmission electron microscopy upon α-synuclein exposure. ER stress was evident by upregulation of GADD34/CHOP, increased phospho-PERK, and reduced nascent protein synthesis. Increased ER–mitochondrial contact via MAMs and enhanced STIM1-ORAI3 clustering reflect compensatory but ultimately insufficient responses to energy stress. Our results reveal that LRRK2-I1371V induces glucose uptake deficits, leading to energy depletion and integrated ER–mitochondria–lysosome dysfunction, thus indicating restoration of astrocytic metabolic capacity as a potential therapeutic strategy for LRRK2-associated PD.

## 1. Introduction

Parkinson’s disease (PD) is a progressive neurodegenerative disorder characterized by the selective degeneration of dopaminergic neurons [1], the accumulation of α-synuclein aggregates [2], and widespread glial dysfunction [3,4]. Among the known genetic contributors, mutations in leucine-rich repeat kinase 2 (LRRK2) account for approximately 5–13% of familial and 1–5% of sporadic PD cases [5]. As in idiopathic PD, brains from LRRK2-mutant PD subjects exhibit significant dopaminergic neurodegeneration in the substantia nigra, accompanied by α-synuclein-positive Lewy body pathology [6,7].

LRRK2 encodes a large, multi-domain protein with both kinase and GTPase activities, positioning it as a critical regulatory hub in vesicle trafficking, autophagy, lysosomal function, and cytoskeletal dynamics [8]. Pathogenic mutations cluster primarily within the Roc-COR-kinase region. The most common is G2019S in the kinase domain, which enhances kinase activity [9]. Key mutations within the GTPase (ROC–COR) domain include R1441C/G/H [10,11] and I1371V [12,13,14,15,16,17,18,19,20]. I1371 resides in a deep hydrophobic pocket at the ROC–ROC dimer interface, where the Cδ methyl group of isoleucine completes van der Waals packing, creating a precisely tuned hydrophobic seal essential for proper GTPase cycling [14]. Although GTPase variants produce classical PD symptoms, their clinical manifestations often differ from those associated with kinase domain mutations [21,22,23,24,25]. Current evidence suggests that LRRK2 mutations exert gain-of-function effects through multiple convergent mechanisms, including enhanced kinase activity, disrupted protein interactions, impaired vesicular trafficking, defective autophagy–lysosomal clearance, and altered synaptic function [26,27,28,29,30].

The traditional neuron-centric view of PD has evolved to recognize the critical contributions of glial cells, particularly astrocytes, to disease pathogenesis and progression [31]. While LRRK2 has been extensively studied in dopaminergic neurons, it is also expressed in astrocytes [32,33], where it influences glutamate uptake and metabolism [34,35], Nrf2-mediated antioxidant systems [34], neurotrophic factor production [36], inflammatory cytokine release [36], and ATP generation [34]. Notably, a single study of LRRK2 G2019S astrocytes reported no significant differences in glucose transporter expression or glucose uptake [31], leaving open the question of whether other LRRK2 mutations affect astrocytic glucose metabolism.

This question is particularly salient given the brain’s extraordinarily high energy demands—consuming approximately 20% of the body’s glucose despite representing only 2% of body weight [37]. Astrocytes play a pivotal role in brain energy metabolism through glucose uptake via GLUT1, glycolytic processing, and lactate provision to neurons via the astrocyte–neuron lactate shuttle (ANLS), which supports synaptic transmission and neuronal survival during periods of high energy demand [38,39]. This metabolic partnership is especially critical in the ageing brain and during neurodegeneration, where energy deficits can precipitate cellular dysfunction and death [40]. Indeed, accumulating evidence indicates that metabolic dysfunction is a core feature of PD. Neuroimaging studies reveal reduced glucose metabolism in affected brain regions before motor symptoms, post-mortem analyses demonstrate mitochondrial respiratory chain defects and alterations in glycolytic enzymes [41,42,43], and epidemiological studies link diabetes and metabolic syndrome to increased PD risk [44,45].

LRRK2’s involvement in glucose metabolism extends beyond the brain. In peripheral insulin-sensitive tissues—muscle and adipose tissue—LRRK2 modulates insulin signalling and systemic glucose homeostasis [46,47,48]. In wild-type mice, a high-fat diet markedly increases LRRK2 expression and phosphorylation of its substrates, Rab8A and Rab10 [46]. Pharmacological LRRK2 inhibition enhances insulin-dependent GLUT4 translocation and glucose uptake in adipocytes, indicating that hyperactive kinase activity suppresses insulin-mediated glucose utilization through phosphorylation of Rab8A and Rab10 [46,47]. Consistent with these findings, LRRK2 phosphorylates Rab8A in a glucose-dependent manner, facilitating its recruitment to the primary cilium [49]. Our earlier work demonstrated that the LRRK2-I1371V mutation drives kinase hyperactivity, leading to hyperphosphorylation of Rab8A and Rab10, which control cholesterol and protein trafficking to the membrane, respectively [50,51]. Importantly, our recently accepted study further shows that Rab8A phosphorylation is significantly higher in LRRK2-I1371V-overexpressing cells than in cells harbouring the G2019S kinase domain variant, indicating mutation-specific amplification of Rab8A dysregulation [51]. This finding was also observed in LRRK2-I1371V astrocytes, suggesting that cell surface GLUT1 expression and glucose metabolism may be impaired in these cells.

Impaired glucose metabolism and ATP depletion have profound consequences for organellar function. Mitochondria, particularly vulnerable to glucose deprivation, exhibit loss of membrane potential and elevated reactive oxygen species (ROS) in PD [52,53,54,55,56,57,58,59]. The endoplasmic reticulum (ER), responsible for protein folding, lipid synthesis, and calcium storage, is exquisitely sensitive to cellular energy status; energy depletion compromises ER protein-folding capacity through reduced ATP availability for chaperone function and impaired calcium homeostasis, leading to activation of the unfolded protein response (UPR) [60,61,62,63]. Lysosomes, the primary degradative organelles that clear damaged proteins and organelles through autophagy, are intimately linked to PD pathogenesis through α-synuclein accumulation and impaired clearance [64,65,66]. The energy-intensive nature of lysosomal biogenesis, acidification, and proteolytic activity makes these organelles highly susceptible to ATP depletion [67].

Mounting evidence indicates extensive functional interconnections among mitochondria, ER, and lysosomes—collectively termed the “mitochondria-ER-lysosome axis”—in which dysfunction in one organelle propagates to others through shared metabolic pathways, calcium signalling, and membrane contact sites [68,69,70,71,72]. This integrated dysfunction explains why therapeutic interventions targeting individual organelles have failed to progress or show efficacy in human PD trials, necessitating the identification of upstream targets [73,74,75,76,77,78]. Distinct LRRK2 variants have been associated with diverse organellar stresses, including impaired mitochondrial dynamics [31,79], ER dysfunction [80,81], and defective lysosomal degradation [82,83]. However, most studies have examined these effects in isolation, without considering how variant-specific glucose disruption may propagate across organellar networks.

Despite growing recognition of astrocytic contributions to LRRK2-associated PD, our understanding of how LRRK2 mutations—particularly the I1371V GTPase domain variant—affect astrocytic glucose metabolism and downstream organellar function remains limited. While previous studies have identified metabolic alterations in LRRK2 astrocytes [31], the mechanistic links between glucose uptake deficits, energy failure, and multi-organellar dysfunction remain poorly characterized. This study, therefore, aims to determine how the LRRK2-I1371V mutation alters glucose uptake and metabolism in astrocytes, as well as organellar function, including mitochondrial health, ER calcium homeostasis, protein synthesis, ER–mitochondrial contact sites (MAMs), and lysosomal degradative capacity. By linking LRRK2-I1371V-driven metabolic deficits to organellar dysfunction, we seek to uncover how astrocytic energy failure contributes to impaired neuronal support and vulnerability in PD, with particular attention to whether this mutation produces distinct metabolic phenotypes compared to the extensively studied G2019S kinase domain variant.

## 2. Results

### 2.1. Disrupted Glucose Handling and Reduced Lactate Release in PD Astrocytes

Astrocytes are the primary regulators of neuronal energy supply. They import glucose predominantly through the transporter GLUT1, and their end-foot processes convert it via glycolysis into lactate and shuttle lactate to neurons through MCT4 to sustain oxidative metabolism [84]. Disruption of this astrocyte–neuron lactate shuttle has been increasingly linked to neurodegeneration in PD [85].

**Figure 1:**
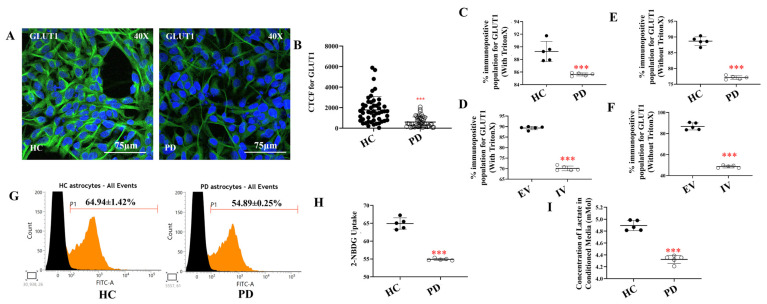
Impaired glucose handling in LRRK2 I1371V astrocytes. (**A**) ICC images of HC and PD astrocytes stained for GLUT1, with nuclei counterstained with DAPI. (**B**) Scatter plot of the CTCF values of the intensity of GLUT1 [*p* < 0.001; HC vs. PD] (data are mean ± SD, two-sample *t*-test; *n* = 50). (**C**,**D**) Graphical representation of the flow cytometry quantified positive population for total GLUT1 in HC and PD astrocytes (**C**) and EV and IV transfected U87 cells (**D**). (Data are mean ± SD, two-sample *t*-test; *n* = 5). [*p* < 0.001; HC vs. PD; EV vs. IV]. (**E**,**F**) Graphical representation of the flow cytometry quantified positive population for membrane-localized GLUT1 in HC and PD astrocytes (**E**) and EV and IV transfected U87 cells (**F**). (Data are mean ± SD, two-sample *t*-test; *n* = 5). [*p* < 0.001; HC vs. PD; EV vs. IV]. (**G**) Representative flow cytometry histograms of 2-NBDG uptake in HC and PD astrocytes. (**H**) Quantification of mean fluorescence intensity (MFI) from 2-NBDG flow cytometric assay. [*p* < 0.001; HC vs. PD] (data are mean ± SD, two-sample *t*-test; *n* = 5). (**I**) Release of lactate into the conditioned media from HC and PD astrocytes. [*p* < 0.001; HC vs. PD] (data are mean ± SD, two-sample *t*-test; *n* = 5). ***—*p* < 0.001 (HC versus PD or EV versus IV).

To investigate whether glucose metabolism is impaired in I1371V astrocytes, we differentiated iPSCs from a healthy control (HC) and a PD patient carrying the I1371V mutation into astrocytes using our previously published protocol [34]. Immunofluorescence analysis showed significantly reduced GLUT1 expression in PD astrocytes compared with controls (Figure 1A,B; *p* < 0.001). Flow cytometric analysis corroborated this finding, demonstrating a markedly lower percentage of GLUT1-immunopositive cells in the PD astrocyte population relative to HC astrocytes (Figure 1C, Supplementary Figure S3A,B *p* < 0.001). To validate these observations in an independent model system, we transfected U87 cells with either LRRK2 I1371V (IV) or empty vector (EV) controls. Consistent with our iPSC-derived astrocyte data, IV-transfected U87 cells exhibited a significantly lesser GLUT1-immunopositive population compared to EV-transfected controls (Figure 1D, Supplementary Figure S3C,D, *p* < 0.001). Analysis of cell surface GLUT1 expression revealed significantly diminished plasma membrane localization in PD astrocytes compared to HC cells (Figure 1E, Supplementary Figure S1E,F *p* < 0.001), a finding that was reproducibly observed in the U87 transfection model (Figure 1F, Supplementary Figure S3G,H; *p* < 0.001). Since decreased GLUT1 expression is directly linked to compromised glucose transport, we next evaluated 2-NBDG uptake, a fluorescent glucose analogue, using flow cytometry (Figure 1G). PD astrocytes exhibited significantly lower 2-NBDG uptake relative to HC astrocytes (Figure 1H, *p* < 0.001). This reduction in glucose uptake was further underlined by lactate measurements in the conditioned medium, which showed that PD astrocytes released substantially less lactate than their HC counterparts (Figure 1I, *p* < 0.001). Collectively, these findings, along with our earlier report of lesser ATP in PD LRRK2 I1371V astrocytes [34], demonstrate that impaired glucose uptake can be one of the primary reasons for lesser ATP levels.

### 2.2. Loss of Mitochondrial Membrane Potential, Elevated ROS, and Mitophagy Signals in PD Astrocytes

**Figure 2:**
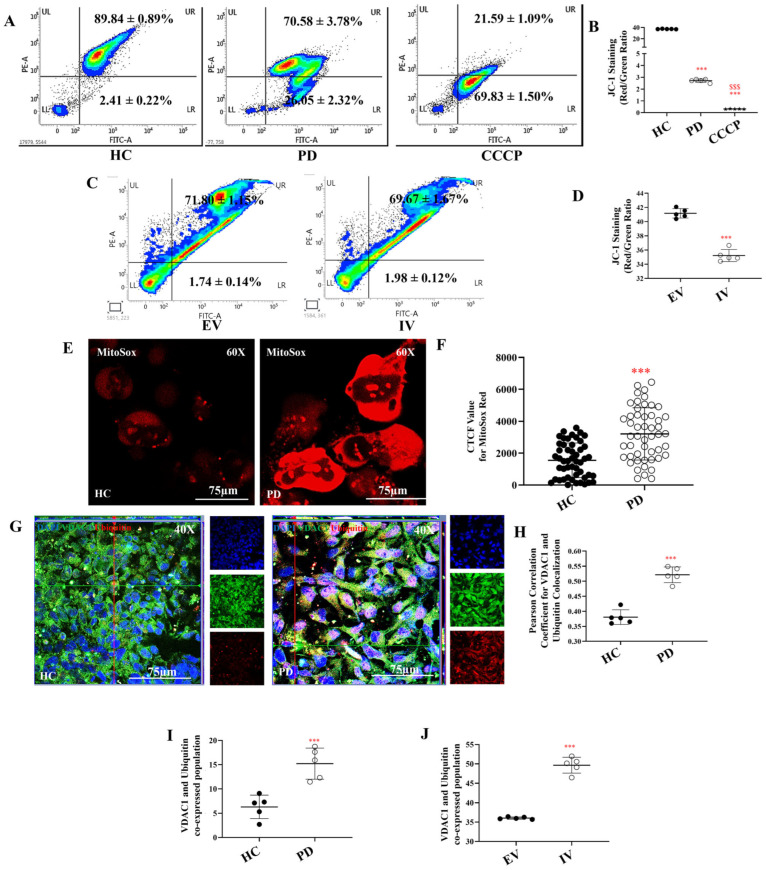
Mitochondrial impairment in LRRK2 I1371V astrocytes. (**A**) Representative JC-1 flow cytometry histograms illustrating red (aggregate) to green (monomer) fluorescence in HC, PD and CCCP-treated positive control astrocytes. (**B**) Quantification of JC-1 aggregate/monomer ratios in HC, PD and CCCP-treated positive control astrocytes [*p* < 0.001; HC vs. PD; CCCP; PD vs. CCCP] (data are mean ± SD, one-way ANOVA; *n* = 5). (**C**) Representative JC-1 flow cytometry histograms illustrating red (aggregate) to green (monomer) fluorescence in EV and IV transfected U87 cells. (**D**) Quantification of JC-1 aggregate/monomer ratios EV and IV transfected U87 cells. [*p* < 0.001; EV vs. IV] (data are mean ± SD, two-sample *t*-test; *n* = 5). (**E**) Representative confocal images of cells stained with MitoSOX Red to detect mitochondrial ROS levels in HC and PD astrocytes. (**F**) Scatter plot of the CTCF values of the intensity of MitoSOX Red [*p* < 0.001; HC vs. PD] (data are mean ± SD, two-sample *t*-test; *n* = 50). (**G**) ICC images of VDAC1 (Alexa Fluor^®^ 488; green) and Ubiquitin (Alexa Fluor^®^ 647; red) coimmunostained HC and PD astrocytes; nucleus counterstained with DAPI. (**H**) Pearson’s coefficient of the colocalization of VDAC1 with Ubiquitin. [*p* < 0.001; HC vs. PD] (data are mean ± SD, two-sample *t*-test; *n* = 5). (**I**,**J**) Graphical representation of percentage of co-positive population of VDAC1 with Ubiquitin in HC and PD astrocytes (**I**) and EV and IV transfected U87 cells (**J**). [*p* < 0.001; HC vs. PD; EV vs. IV] (data are mean ± SD, two-sample *t*-test; *n* = 5). ***—*p* < 0.001 (HC versus PD, CCCP or EV versus IV) $$$—*p* < 0.001 (PD versus CCCP).

The other contributory factor to the lower ATP levels [86] may be mitochondrial impairment, as ATP—the final product of glucose metabolism—is primarily generated within mitochondria. We assessed mitochondrial membrane potential using the ratiometric JC-1 dye, which measures the red-to-green fluorescence ratio. Flow cytometry analysis demonstrated that healthy control (HC) astrocytes exhibited a significantly higher red/green fluorescence ratio compared to PD astrocytes, indicating more intact mitochondrial membrane potential (Figure 2A,B). Representative event-scatter plots are shown in Figure 2A, with the corresponding quantitative analysis presented in Figure 2B. Treatment with CCCP caused a marked decrease in the red/green ratio in HC astrocytes, confirming the assay’s validity.

We obtained similar results in U87 cells transfected with LRRK2 I1371V versus empty vector (EV) controls. FACS analysis revealed that cells expressing the I1371V variant displayed significantly reduced mitochondrial membrane potential compared to EV controls (Figure 2C,D). To ensure reproducibility, we repeated the JC-1 assay using an independent iPSC-derived astrocyte line from a second healthy control (HC02), yielding comparable results (Supplementary Figure S4A,B).

We next investigated whether the mutation also affected mitochondrial oxidative stress. Using MitoSOX Red, we observed markedly elevated fluorescence in PD astrocytes compared to HC cells, indicating increased mitochondrial ROS levels (Figure 2E). Corrected total cell fluorescence (CTCF) analysis confirmed a significant increase in mitochondrial ROS in PD astrocytes (*p* < 0.001, Figure 2F).

Given that mitochondria in PD astrocytes experience significant stress, we examined whether these compromised organelles were being targeted for clearance through mitophagy—a critical protective mechanism for maintaining cellular homeostasis. To confirm that mitochondria in PD astrocytes were indeed stressed and primed for clearance, we assessed ubiquitin recruitment to these organelles. Co-localization analysis of ubiquitin (Ubq) with VDAC1, an outer mitochondrial membrane marker, revealed increased yellow puncta in PD astrocytes (Figure 2G), indicating enhanced mitochondrial ubiquitination. Pearson’s coefficient analysis substantiated this observation, showing significantly higher Ubq–VDAC1 co-localization in PD cells (Figure 2H; *p* < 0.001). Flow cytometric analysis corroborated these findings, with PD astrocytes displaying a significantly higher proportion of co-labelled cells compared to HC cells (Figure 2I, Supplementary Figure S4C,D; *p* < 0.001). LRRK2 I1371V-transfected cells recapitulated these results, exhibiting markedly increased Ubq–VDAC1 co-labelling compared to EV controls (Figure 2J, Supplementary Figure S4E,F; *p* < 0.001).

**Figure 3:**
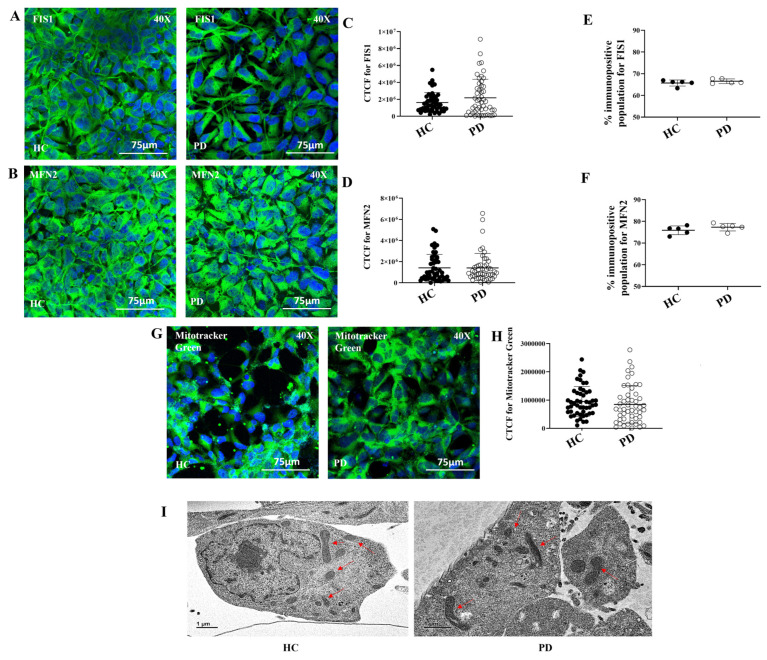
Mitochondrial fission–fusion dynamics and ultrastructure are preserved in LRRK2 I1371V astrocytes. ICC images of HC and PD astrocytes stained for Fis1 (**A**) and Mfn2 (**B**), with nuclei counterstained with DAPI. (**C**,**D**) Scatter plot of the CTCF values of the intensity Fis1 (**C**) and Mfn2 (**D**) [*p* > 0.05; HC vs. PD] (data are mean ± SD, two-sample *t*-test; *n* = 50). (**E**,**F**) Graphical representation of the flow cytometry quantified positive population for Fis1 (**E**) and Mfn2 (**F**) in HC and PD astrocytes [*p* > 0.05; HC vs. PD] (data are mean ± SD, two-sample *t*-test; *n* = 5). (**G**) ICC images of HC and PD astrocytes stained Mitotracker Green, with nuclei counterstained with DAPI. (**H**) Scatter plot of the CTCF values of the intensity of Mitotracker Green [*p* > 0.05; HC vs. PD] (data are mean ± SD, two-sample *t*-test; *n* = 50). (**I**) Transmission electron microscopy (TEM) images depict mitochondrial ultrastructure following 24 h treatment with wild-type α-synuclein in HC and PD astrocytes. Red arrows indicate mitochondria.

We next asked whether mitochondrial stress in PD astrocytes was linked to changes in mitochondrial dynamics. To address this, we examined key proteins involved in mitochondrial fission and fusion. Immunofluorescence staining of the fission protein FIS1 (Figure 3A) and the fusion protein MFN2 (Figure 3B) showed similar patterns in HC and PD astrocytes. Quantification using CTCF revealed no significant differences in protein levels (Figure 3C,D; *p* > 0.05). Flow cytometry confirmed these findings, with no detectable differences between groups (Figure 3E,F; Supplementary Figure S4G–J; *p* > 0.05).

With mitochondrial dynamics unchanged, we next examined mitochondrial mass. Cells were stained with MitoTracker Green (Figure 3G). Quantitative analysis showed no difference in fluorescence intensity between HC and PD astrocytes (Figure 3H; *p* > 0.05). This indicated that mitochondrial mass was preserved. Ultrastructural analysis was performed using TEM. Mitochondria in HC and PD astrocytes appeared similar. No significant changes in morphology or cristae organization were observed (Figure 3I).

### 2.3. Proteasomal Dysfunction and Lysosomal Deficits in I1371V Astrocytes

**Figure 4:**
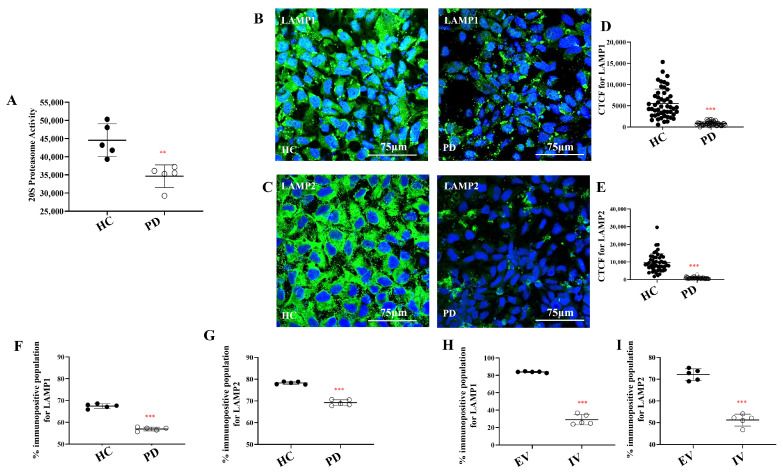
Decreased proteasomal activity and reduction in the number of lysosomes in LRRK2 I1371V astrocytes. (**A**) Quantification of 20S proteasome activity in HC and PD astrocytes [*p* < 0.01; HC vs. PD] (data are mean ± SD, two-sample *t*-test; *n* = 5). (**B**,**C**) ICC images of HC and PD astrocytes stained for LAMP1 (**B**) and LAMP2 (**C**), with nuclei counterstained with DAPI. (**D**,**E**) Scatter plot of the CTCF values of the intensity LAMP1 (**D**) and LAMP2 (**E**) [*p* < 0.001; HC vs. PD] (data are mean ± SD, two-sample *t*-test; *n* = 50). (**F**,**G**) Graphical representation of the flow cytometry quantified positive population for LAMP1 (**F**) and LAMP2 (**G**) in HC and PD astrocytes [*p* < 0.001; HC vs. PD] (data are mean ± SD, two-sample *t*-test; *n* = 5). (**H**,**I**) Graphical representation of the flow cytometry quantified positive population for LAMP1 (**H**) and LAMP2 (**I**) in EV and IV transfected U87 cells [*p* < 0.001; EV vs. IV] (data are mean ± SD, two-sample *t*-test; *n* = 5). **—*p* < 0.01 (HC versus PD); ***—*p* < 0.001 (HC versus PD or EV versus IV).

Given the compromised mitochondrial function and reduced ATP synthesis, we investigated how this metabolic vulnerability affects protein degradation—a highly energy-dependent process. We examined the proteasome, which plays an essential role in degrading ubiquitinated mitochondria during mitophagy. Assessment of 20S proteasomal activity revealed significant reduction in PD astrocytes compared to HCs (Figure 4A; *p* < 0.01). These findings indicate that although dysfunctional mitochondria in PD astrocytes are appropriately tagged for clearance, impaired proteasomal activity prevents their efficient removal, thereby contributing to the persistence of stressed organelles and exacerbating cellular dysfunction.

Following the identification of proteasomal impairment, we examined the lysosomal degradation pathway, which represents the final step in autophagic clearance. Immunocytochemistry revealed reduced expression of lysosomal markers LAMP1 and LAMP2 in PD astrocytes compared to HCs (Figure 4B,C). Quantitative corrected total cell fluorescence (CTCF) analysis confirmed significant decreases in both markers (Figure 4D,E; *p* < 0.001). Flow cytometry validated these findings, demonstrating significantly lower LAMP1 and LAMP2 expression in PD astrocytes (Figure 4F,G, Supplementary Figure S5A–D; *p* < 0.001). Importantly, no significant differences were observed between the two independent healthy control astrocyte lines (HC and HC02) (Supplementary Figure S5E–H), confirming the mutation-specific nature of these changes. Supporting this conclusion, I1371V-transfected U87 cells similarly exhibited reduced LAMP1 and LAMP2 expression compared to empty vector controls (Figure 4H,I, Supplementary Figure S5I–L; *p* < 0.001).

**Figure 5:**
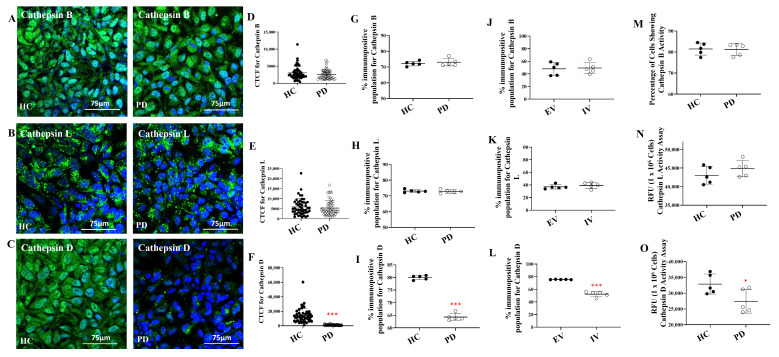
Selective reduction cathepsin D levels and activity in LRRK2 I1371V astrocytes. (**A**–**C**) ICC images of HC and PD astrocytes stained for cathepsin B (**A**), cathepsin L (**B**) and cathepsin D (**C**) with nuclei counterstained with DAPI. (**D**,**F**) Scatter plot of the CTCF values of the intensity cathepsin B (**D**), cathepsin L (**E**) and cathepsin D (**F**) [*p* < 0.001; HC vs. PD] (data are mean ± SD, two-sample *t*-test; *n* = 50). (**G**–**I**) Graphical representation of the flow cytometry quantified positive population for cathepsin B (**G**), cathepsin L (**H**) and cathepsin D (**I**) in HC and PD astrocytes (**G**) [*p* < 0.001; HC vs. PD] (data are mean ± SD, two-sample *t*-test; *n* = 5). (**J**–**L**) Graphical representation of the flow cytometry quantified positive population for cathepsin B (**J**), cathepsin L (**K**) and cathepsin D (**L**) in EV and IV transfected U87 cells (**I**) [*p* < 0.001; EV vs. IV] (data are mean ± SD, two-sample *t*-test; *n* = 5). (**M**) cathepsin B Activity in HC and PD astrocytes. (data are mean ± SD, two-sample *t*-test; *n* = 5). (**N**) Cathepsin L Activity in HC and PD astrocytes. (Data are mean ± SD, two-sample *t*-test; *n* = 5). (**O**) Cathepsin D Activity in HC and PD astrocytes. (Data are mean ± SD, two-sample *t*-test; *n* = 5). *—*p* < 0.05 (HC versus PD); ***—*p* < 0.001 (HC versus PD or EV versus IV).

To further investigate lysosomal functionality, we examined the expression of key lysosomal proteases: cathepsins B, L, and D. ICC analysis revealed no observable differences in fluorescence intensity for cathepsins B and L between PD and HC astrocytes (Figure 5A,B). In contrast, cathepsin D fluorescence was significantly reduced in PD astrocytes (Figure 5C). CTCF quantification confirmed these observations, showing no significant differences for cathepsins B and L between groups (Figure 5D,E, *p* > 0.05), while cathepsin D levels were significantly lower in PD astrocytes (Figure 5F; *p* < 0.001). Flow cytometry corroborated these findings, with cathepsins B and L expression remaining comparable between HC and PD groups (Figure 5G,H, Supplementary Figure S6A–D; *p* > 0.05), whereas cathepsin D expression was significantly decreased in PD astrocytes (Figure 5I, Supplementary Figure S6E,F; *p* < 0.001). Parallel analysis of EV and IV cells yielded identical results, highlighting reduced cathepsin D expression relative to EV controls (Figure 5J–L, Supplementary Figure S6G–L; *p* < 0.001).

To determine whether these expression changes translated to functional differences, we assessed enzymatic activities of these cathepsins. Flow cytometry-based activity assays for cathepsin B and spectrofluorometric assays for cathepsin L revealed comparable enzymatic activities between healthy control and PD astrocytes, with no statistically significant differences observed (Figure 5M,N; Supplementary Figure S6M,N; *p* > 0.05). In stark contrast, functional assessment of cathepsin D enzymatic activity revealed a significant reduction in PD astrocytes compared to healthy controls (Figure 5O; *p* < 0.05), demonstrating that both cathepsin D expression and its proteolytic function are compromised in these cells.

**Figure 6:**
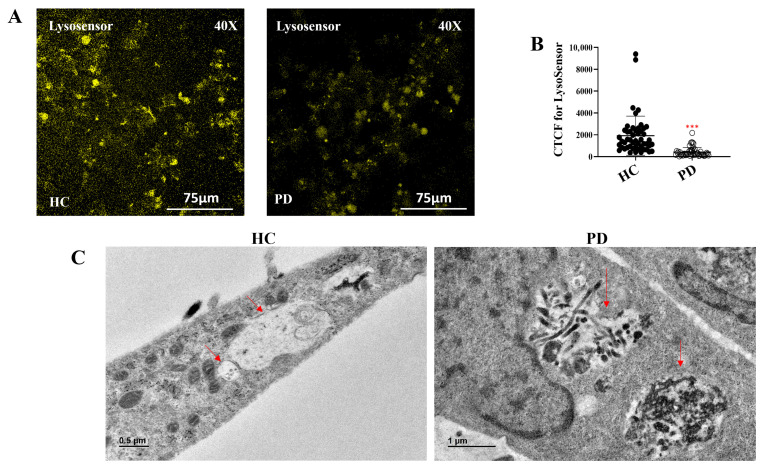
Altered lysosomal pH and dense lysosomal accumulation upon α-Synuclein treatment in LRRK2 I1371V astrocytes. (**A**) Representative confocal images of LysoSensor staining showing lysosomal pH in healthy control (HC) and LRRK2 I1371V Parkinson’s disease (PD) astrocytes. (**B**) Scatter plot of the CTCF values of the intensity of Lysosensor [*p* < 0.001; HC vs. PD] (data are mean ± SD, two-sample *t*-test; *n* = 50). (**C**) Transmission electron microscopy (TEM) images depict lysosomal ultrastructure following 24 h treatment with wild-type α-synuclein. Red arrows indicate lysosomes. PD astrocytes display densely packed, electron-dense lysosomes, reflecting an accumulation of undigested material, whereas HC astrocytes exhibit normal lysosomal morphology. ***—*p* < 0.001 (HC versus PD).

To gain additional insight into lysosomal function, we assessed lysosomal pH using LysoSensor-based immunofluorescence imaging. HC astrocytes displayed robust LysoSensor fluorescence, while PD astrocytes exhibited significantly reduced signal intensity (Figure 6A,B; *p* < 0.001), consistent with impaired acidification. Since acidic pH is essential for lysosomal protease function, this finding suggested compromised degradative capacity. To test this hypothesis, we exposed astrocytes to wild-type monomeric α-synuclein for 24 h and examined their ultrastructure using transmission electron microscopy (TEM). HC astrocytes displayed lysosomes containing clearly degraded material, whereas PD astrocytes showed numerous lysosomes packed with undegraded vesicular content (Figure 6C).

These findings demonstrate that the LRRK2 I1371V mutation compromises lysosomal integrity in PD astrocytes. The combination of reduced lysosome number, impaired acidification, and diminished cathepsin D activity collectively hinders autophagy. Consequently, damaged mitochondria and undegraded cargo accumulate, perpetuating mitochondrial stress and driving astrocytic dysfunction.

### 2.4. Impaired Calcium Dynamics in LRRK2 I1371V Astrocytes

**Figure 7:**
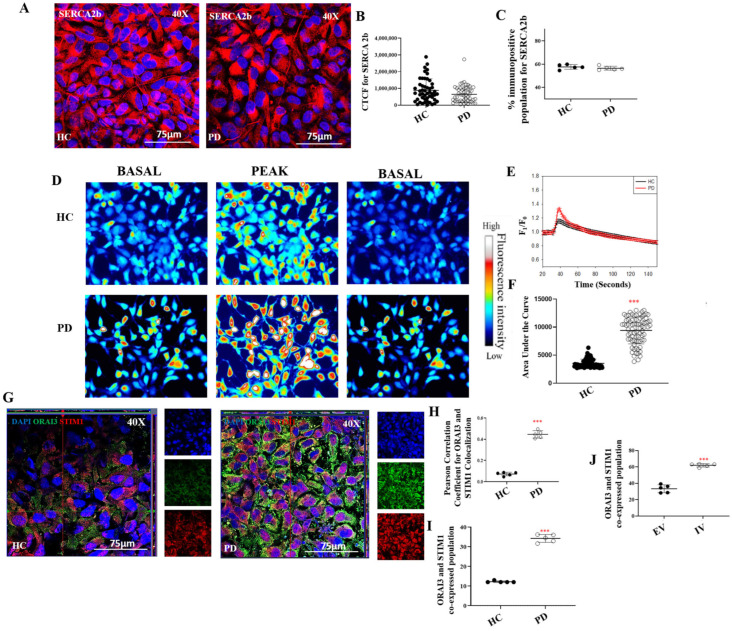
Impairment in the calcium dynamics of LRRK2 I1371V astrocytes. ICC images of HC and PD astrocytes stained for SERCA2 (**A**) with nuclei counterstained with DAPI. (**B**) Scatter plot of the CTCF values of the intensity for SERCA2 [*p* > 0.05; HC vs. PD] (Data are mean ± SD, two-sample *t*-test; *n* = 50). (**C**) Graphical representation of the flow cytometry quantified positive population for SERCA2 (**C**) in HC and PD astrocytes [*p* > 0.05; HC vs. PD] (data are mean ± SD, two-sample *t*-test; *n* = 5). (**D**) ICC images of ORAI3 (Alexa Fluor^®^ 488; green) and STIM1 (Alexa Fluor^®^ 647; red) coimmunostained HC and PD astrocytes; nucleus counterstained with DAPI. (**E**) Pearson’s coefficient of the colocalization of ORAI3 with STIM1. [*p* < 0.001; HC vs. PD] (data are mean ± SD, Two Sample *t*-test; *n* = 5) (**F**,**G**) Graphical representation of percentage of co-positive population of ORAI3 with STIM1 in HC and PD astrocytes (**F**) and EV and IV transfected U87 cells (**G**). [*p* < 0.001; HC vs. PD; EV vs. IV] (data are mean ± SD, two-sample *t*-test; *n* = 5). (**H**) Representative fluorescence images of Fluo4-loaded HC and PD astrocytes at various time points, captured before and after the addition of 2mM calcium. (**I**) Represents the mean normalized fluorescence intensity of the Fluo4-labelled HC and LRRK2-I1371V PD astrocytes on stimulation with α-synuclein aggregates. (**J**) Represents the area under the curve of [Ca^2+^]i response shown in panels (**H**) for the HC and LRRK2-I1371V PD astrocytes. [*p* < 0.001; HC vs. PD]. ***—*p* < 0.001 (HC versus PD or EV versus IV).

Given that ATP drives SERCA2-mediated Ca^2+^ transport from the cytosol to the ER, we assessed SERCA expression. Immunofluorescence analysis revealed no significant difference in SERCA2 levels between PD and healthy control (HC) astrocytes (Figure 7A), which was further confirmed by CTCF quantification (Figure 7B; *p* > 0.05). Flow cytometry analysis corroborated these findings, showing no alteration in SERCA2 protein expression between the two groups (Figure 7C; Supplementary Figure S7A,B; *p* > 0.05). However, given the earlier reduction in ATP levels in PD astrocytes [34], SERCA2 function is likely compromised despite unchanged expression, as insufficient ATP availability would impair its calcium-pumping capacity, thereby depleting ER Ca^2+^ stores.

Depletion of ER Ca^2+^ stores triggers STIM1 activation and clustering at ER–plasma membrane contact sites, where it interacts with ORAI channels to initiate store-operated Ca^2+^ entry (SOCE). To investigate whether this compensatory mechanism is activated in I1371V astrocytes, we examined the membrane distribution of STIM1 and ORAI3 by immunofluorescence without permeabilization, thereby visualizing only plasma membrane-localized proteins. Orthogonal projections revealed a striking increase in ORAI3–STIM1 co-localisation on the astrocyte membrane in PD cells compared to HCs, evident as prominent yellow puncta in the merged channels (Figure 7D). Quantification using Pearson’s coefficient confirmed significantly higher membrane association of these proteins in PD astrocytes (Figure 7E; *p* < 0.001). Flow cytometry further supported these findings, demonstrating increased surface co-labelling of ORAI3 and STIM1 in PD astrocytes relative to HC (Figure 7F; Supplementary Figure S7C,D; *p* < 0.001). Similarly, U87 cells transfected with the LRRK2 I1371V construct (IV) displayed significantly higher membrane co-localisation of ORAI3 and STIM1 compared to empty vector (EV) controls (Figure 7G; Supplementary Figure S5E,F; *p* < 0.001), confirming that the LRRK2 mutation directly drives this effect.

To assess the functional consequences of enhanced STIM1–ORAI3 coupling, we measured SOCE responses using Fluo-4 AM calcium imaging. Representative fluorescence images captured before and after extracellular calcium addition are shown in Figure 7H. PD astrocytes exhibited a significantly elevated SOCE response compared to HCs (Figure 7H,I). Normalized fluorescence intensity (F_1_/F_0_) was plotted over time to compare calcium dynamics between groups (Figure 7I), and analysis of the area under the curve confirmed significantly higher calcium influx in PD astrocytes relative to HC (Figure 7J; *p* < 0.001). These findings demonstrate that reduced glucose uptake and ATP levels in LRRK2 I1371V astrocytes impair SERCA2-mediated ER calcium transport, with Ca^2+^ depletion triggering compensatory upregulation of STIM1 and ORAI3 juxtaposition and leading to increased SOCE.

### 2.5. LRRK2 I1371V Enhances ER–Mitochondria Contact via MAMs

**Figure 8:**
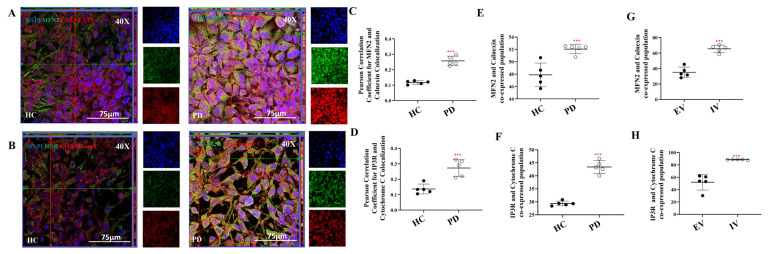
LRRK2 I1371V promotes increased ER–mitochondria association at MAMs. (**A**) ICC images of MFN2 (Alexa Fluor^®^ 488; green) and Calnexin (Alexa Fluor^®^ 647; red) coimmunostained HC and PD astrocytes; nucleus counterstained with DAPI. (**B**) ICC images of IP3R (Alexa Fluor^®^ 488; green) and Cytochrome C (Alexa Fluor^®^ 647; red) coimmunostained HC and PD astrocytes; nucleus counterstained with DAPI. (**C**,**D**) Pearson’s coefficient of the colocalization of MFN2 with calnexin (**C**) and IP3R and ccytochrome C (**D**) [*p* < 0.001; HC vs. PD] (data are mean ± SD, two-sample *t*-test; *n* = 5). (**E**,**F**) Graphical representation of percentage of co-positive population of MFN2 with calnexin (**E**) and IP3R and cytochrome C (**F**) in HC and PD astrocytes. (**G**,**H**) Graphical representation of percentage of co-positive population of MFN2 with calnexin (**G**) and IP3R and cytochrome C (**H**) in EV and IV transfected U87 cells. [*p* < 0.001; HC vs. PD; EV vs. IV] (data are mean ± SD, two sample *t*-test; *n* = 5). ***—*p* < 0.001 (HC versus PD or EV versus IV).

Given the pronounced alterations in ER Ca^2+^ handling observed in LRRK2 I1371V–expressing cells, we next investigated whether ER–mitochondria contact sites, or mitochondria-associated membranes (MAMs), were correspondingly altered. MAM formation was assessed by immunofluorescence-based co-localisation analysis of established ER and mitochondrial markers. Specifically, we examined the spatial association of the mitochondrial proteins MFN2 and cytochrome C with the ER proteins calnexin and IP3R, respectively. Orthogonal projections revealed a pronounced increase in co-localisation of both protein pairs in PD astrocytes, evident as prominent yellow puncta in merged images (Figure 8A,B).

Quantitative analysis using Pearson’s correlation coefficient demonstrated a significantly higher degree of co-localisation between MFN2–calnexin (Figure 8C; *p* < 0.001) and IP3R–cytochrome C (Figure 8D; *p* < 0.001) in PD astrocytes compared to healthy controls (HCs). Consistent with these findings, flow cytometric analysis revealed a significantly greater proportion of PD astrocytes co-labelled for MFN2 and calnexin (Figure 8E; Supplementary Figure S8A,B; *p* < 0.001), as well as IP3R and cytochrome C (Figure 8F; Supplementary Figure S8C,D; *p* < 0.001), relative to HC.

To determine whether these alterations were directly attributable to the LRRK2 I1371V mutation, parallel experiments were performed in U87 cells transfected with empty vector (EV) or LRRK2 I1371V (IV). IV-expressing cells recapitulated the phenotype observed in PD astrocytes, exhibiting significantly increased co-localisation of MFN2–calnexin and IP3R–cytochrome C compared to EV controls (Figure 8G,H; Supplementary Figure S8E–H; *p* < 0.001).

Together, these results indicate that the LRRK2 I1371V mutation promotes ER–mitochondria coupling by increasing MAM formation. Such augmented ER–mitochondrial crosstalk is likely to facilitate excessive calcium transfer to mitochondria, thereby contributing to the mitochondrial dysfunction observed in PD astrocytes.

### 2.6. ER Stress and Impaired Protein Synthesis in LRRK2 I1371V Astrocytes

**Figure 9:**
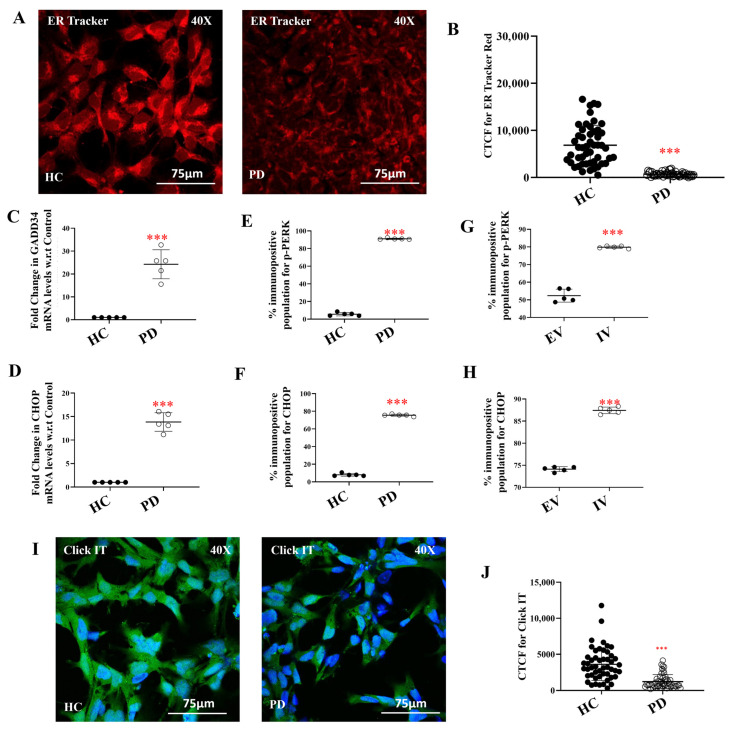
Endoplasmic reticulum stress and disrupted protein synthesis in LRRK2 I1371V astrocytes. (**A**) Representative confocal images showing ER morphology using methyl ER-Tracker™ Red (BODIPY™ TR Glibenclamide) staining in healthy control (HC) and LRRK2 I1371V Parkinson’s disease (PD) astrocytes. (**B**) Scatter plot of the CTCF values of the intensity of ER-Tracker [*p* < 0.001; HC vs. PD] (data are mean ± SD, two-sample *t*-test; *n* = 50). (**C**,**D**) qPCR. (**C**,**D**) Quantification of mRNA gene expression of GADD34 (**C**) and CHOP (**D**) in HC and PD astrocytes using quantitative polymerase chain reaction (qPCR) [*p* < 0.001; HC vs. PD] (Data are mean ± SD, two-sample *t*-test; *n* = 50). (**E**,**F**) Graphical representation of the flow cytometry quantified positive population for p-PERK (**E**) and CHOP (**F**) in HC and PD astrocytes [*p* < 0.001; HC vs. PD] (data are mean ± SD, two-sample *t*-test; *n* = 5). (**G**,**H**) Graphical representation of the flow cytometry quantified positive population for p-PERK (**G**) and CHOP (**H**) in EV and IV transfected U87 cells [*p* < 0.001; EV vs. IV] (data are mean ± SD, two-sample *t*-test; *n* = 5). (**I**) Representative Click-iT™ HPG Alexa Fluor™ 488 images show newly synthesized proteins (green) with nuclei counterstained with DAPI in HC and PD astrocytes. (**J**) Scatter plot of the CTCF values of the intensity of Click-iT™ HPG Alexa Fluor™ 488 [*p* < 0.001; HC vs. PD] (data are mean ± SD, two-sample *t*-test; *n* = 50). ***—*p* < 0.001 (HC versus PD or EV versus IV).

Energy deficits arising from mitochondrial dysfunction and impaired lysosomal clearance are expected to hamper energy-intensive cellular processes, including protein synthesis. Building on the evidence of mitochondrial depolarization and oxidative stress, the LRRK2 I1371V mutation was hypothesized to perturb ER function, given its central role in protein folding, calcium homeostasis, and organelle crosstalk. To assess ER integrity, astrocytes were stained with ER-Tracker dye. PD astrocytes exhibited significantly reduced fluorescence intensity compared to healthy controls (HCs), suggesting ER disruption and elevated stress (Figure 9A). Quantification using CTCF confirmed a significant reduction in ER signal in PD cells (Figure 9B; *p* < 0.001). ER stress was further examined at the transcriptional level by measuring the expression of key unfolded protein response (UPR) genes. Both GADD34 and CHOP genes were significantly upregulated in PD astrocytes in comparison to HC, leading to activation of the ER stress response (Figure 9C,D; *p* < 0.001). At the protein level, phosphorylated PERK (*p*-PERK) and CHOP levels were assessed using flow cytometry. Both markers were significantly higher in PD astrocytes compared to HCs (Figure 9E,F, Supplementary Figure S9A–D; *p* < 0.001). To ensure that these differences were mutation-specific and not due to variability among control lines, a second healthy iPSC-derived astrocyte line (HC02) was analyzed, which showed no significant difference in expression compared to the original HC line (Supplementary Figure S9E–H). Similar upregulation of p-PERK and CHOP was observed in U87 cells transfected with EV and IV, with IV cells showing significantly higher levels of both markers (Figure 9G–H, Supplementary Figure S9I–L; *p* < 0.001). This confirmed that the LRRK2 I1371V mutation directly contributes to ER stress activation. Because chronic ER stress impairs protein synthesis, nascent protein production was evaluated using the Click-iT assay. Consistent with elevated stress levels, PD astrocytes exhibited a significant reduction in protein synthesis relative to HC, as quantified by CTCF (Figure 9I,J; *p* < 0.001), further supporting ER dysfunction in LRRK2 mutant astrocytes.

## 3. Discussion

We report the first comprehensive analysis of glucose metabolism and organellar dysfunction in LRRK2 I1371V astrocytes. Our findings reveal a primary metabolic vulnerability: reduced GLUT1 expression and membrane localisation lead to impaired glucose uptake, decreased lactate production, and ATP depletion. This metabolic insufficiency triggers cascading dysfunction across mitochondria, lysosomes, and the endoplasmic reticulum (ER), thereby establishing how GTPase domain mutations in LRRK2 drive astrocytic energy failure.

The brain depends almost exclusively on glucose for energy, with astrocytes orchestrating supply through GLUT1-mediated glucose uptake, glycolytic lactate production, and MCT4-mediated lactate export to neurons [84]. Disruption of this astrocyte–neuron lactate shuttle contributes to neurodegeneration in PD [87], leaving neurons vulnerable to energy depletion and oxidative stress when astrocytic lactate supply is compromised. Both astrocytic glycolysis and overall brain glucose metabolism decline with age and in neurodegenerative conditions [38]. Patient-derived I1371V astrocytes showed significantly reduced GLUT1 expression—both total and plasma membrane-localized—confirmed independently in U87 cells expressing this variant. Reduced 2-NBDG uptake and decreased lactate release indicate substantially impaired glucose transport and glycolytic flux, with concomitant downregulation of LDH and MCT4 transcripts pointing to collapse of the metabolic pathway coupling glycolysis to neuronal energy support.

This metabolic phenotype distinguishes I1371V from the well-characterized G2019S kinase domain mutation. iPSC-derived G2019S astrocytes show no GLUT1 mRNA deficits, glucose uptake impairment, or ATP synthesis abnormalities [31,83], contrasting sharply with the reduced ATP production we documented in I1371V astrocytes [34]. This divergence indicates that distinct LRRK2 mutations differentially impact astrocytic energy metabolism, with I1371V creating a unique vulnerability through dysfunction of the GTPase domain.

Multiple converging mechanisms likely drive reduced GLUT1 expression and membrane localization in I1371V astrocytes. LRRK2 regulates vesicular trafficking through phosphorylation of Rab GTPases, particularly Rab8A and Rab10, which control membrane protein trafficking and recycling [50,88,89]. Our previous work demonstrated hyperphosphorylation of Rab8A and Rab10 in I1371V astrocytes [34,50], and disrupting membrane cholesterol trafficking and receptor localization [85,90,91,92,93,94,95,96,97,98,99,100,101,102,103]. Notably, in our recently accepted study, Rab8A phosphorylation was significantly higher in LRRK2 I1371V-overexpressing cells than in cells harbouring the kinase domain G2019S variant, indicating variant-specific amplification of Rab8A dysregulation [51]. Given that Rab8A regulates GLUT4 translocation in adipocytes [46] and LRRK2 phosphorylates Rab8A in a glucose-dependent manner to regulate trafficking [93], aberrant Rab8A hyperphosphorylation in I1371V astrocytes likely disrupts GLUT1 trafficking to the plasma membrane. Our observation of reduced cell surface GLUT1 despite residual total cellular expression supports impaired transporter trafficking rather than synthesis failure. Rab8A phosphorylation was, in particular, higher for the LRRK2 I1371V overexpressed cells than G2019S [51].

The severe mitochondrial dysfunction observed—reduced membrane potential, elevated ROS, and increased ubiquitination—directly follows glucose deprivation and ATP depletion. Both iPSC-derived I1371V astrocytes and U87 cells expressing this variant showed reduced JC-1 red-to-green fluorescence ratios, indicating impaired oxidative phosphorylation [94]. Similar defects occur in other pathogenic LRRK2 variants, including G2019S [85,95,96,97,98,99] and R1441C [100], though their relationship to glucose metabolism remains underexplored. Elevated mitochondrial ROS likely reflects a compromised NADPH supply from the glucose-dependent pentose phosphate pathway [101,102]. Although mitochondria are appropriately tagged for removal (enhanced ubiquitin-VDAC1 colocalization), we acknowledge that these co-localisation findings represent proximity associations within Airyscan (~120–140 nm lateral resolution)-limited resolution; future studies employing super-resolution microscopy platforms—such as STORM, STED—would provide nanoscale validation of these spatial relationships. Reduced ATP-dependent proteasomal activity [103] would impair clearance. Minimal alterations in mitochondrial mass and fission–fusion balance indicate early functional stress rather than overt structural damage, suggesting that interventions bypassing glucose uptake deficits—such as ketone bodies or direct lactate administration—could ameliorate mitochondrial dysfunction.

ATP depletion extends beyond energy shortages to dismantle cellular quality control. The comprehensive lysosomal deficits in I1371V astrocytes—reduced LAMP1/LAMP2 expression, impaired acidification, diminished cathepsin D expression and activity, and accumulation of undegraded cargo—represent critical failures in proteostasis directly relevant to PD pathogenesis. cathepsin D serves as the primary protease for lysosomal α-synuclein degradation [104,105], and its mutations cause neuronal ceroid lipofuscinosis and Parkinsonism [104,105,106,107,108,109,110,111,112,113,114,115,116,117]. Reduced cathepsin D activity appears in sporadic PD patient brains [109], while GBA1 mutations increase α-synuclein levels through effects on lysosomal cathepsin D [110]. Impaired lysosomal acidification mechanistically explains reduced cathepsin D activity, as this aspartic protease requires pH 3.5–5.0 for function [111]. Maintaining lysosomal acidity demands substantial ATP for vacuolar H+-ATPases [112,113,114], making acidification sensitive to cellular energy status. The glucose uptake deficits and reduced ATP production in I1371V astrocytes directly compromise v-ATPase function, alkalinizing lysosomes and inactivating pH-dependent proteases. Transmission electron microscopy revealed lysosomes packed with undegraded vesicular material following α-synuclein exposure—ultrastructural evidence of compromised degradative capacity. This has critical implications: if I1371V astrocytes cannot efficiently degrade internalized α-synuclein, they may become sources of pathological protein propagation rather than protective clearance [115,116,117]. The transcription factor EB (TFEB), a master regulator of lysosomal biogenesis, requires energy-dependent nuclear translocation and is inhibited under energy stress [118,119], which explains reduced LAMP1/LAMP2 expression in metabolically compromised I1371V astrocytes.

Energy deficits also impaired ER function. ATP drives SERCA2ab-mediated Ca^2+^ transport from the cytosol to the ER, which is essential for protein synthesis [120]. I1371V astrocytes showed increased Stim1-Orai3 juxtaposition, indicating ER Ca2+ depletion, elevated cytosolic Ca^2+^ via store-operated calcium entry (SOCE), and inadequate ER refilling despite normal SERCA2ab expression—consistent with ATP insufficiency impairing SERCA2ab function. Nevertheless, contributions from other ER calcium regulatory components—including inositol 1,4,5-trisphosphate receptors (IP3Rs), ryanodine receptors, or calreticulin-mediated buffering—cannot be entirely ruled out, and exploring their respective roles in LRRK2 I1371V astrocytic calcium dysregulation represents a promising avenue for future research. Simultaneously, increased ER–mitochondrial contact via MAMs may represent a compensatory enhancement of Ca^2+^ transfer to boost mitochondrial ATP production [121,122]. Whereas the endoplasmic reticulum and mitochondria have long been recognized as independent nutrient sensors that adapt cellular metabolism, the ER–mitochondria interface (MAMs) has more recently emerged as a nutrient-regulated signalling hub that tunes mitochondrial function to the metabolic state. Studies in pancreatic β-cells [123] and neuronal [124] models have demonstrated that glucose availability dynamically regulates ER–mitochondria contacts and Ca^2+^ coupling; however, such regulation has not been described in astrocytes. Our findings provide the first evidence that, in astrocytes, a pathological metabolic context driven by a mutation in the LRRK2 GTPase domain is associated with increased MAM formation, suggesting that altered ER–mitochondria coupling represents an adaptive—or maladaptive—response to energy stress in these cells.

ER-Tracker staining revealed reduced ER signal in I1371V astrocytes, indicating structural disruption. Activation of ER stress pathways (upregulated GADD34, CHOP, and phosphorylated PERK) with reduced nascent protein synthesis reflects cellular response to energy insufficiency. The ER folds approximately one-third of cellular proteins via ATP-dependent processes [125,126], and energy depletion leads to the accumulation of unfolded proteins and UPR activation. Chronic ER stress inhibits global protein synthesis, as demonstrated by Click-iT assays showing significantly reduced nascent protein production in I1371V astrocytes. Reduced protein synthesis capacity profoundly impacts neuronal support. Astrocytes synthesize and secrete neurotrophic factors, antioxidant proteins, and metabolic enzymes critical for neuronal health [81,127], including GDNF, BDNF, and glutathione—particularly important for dopaminergic neuron survival [34,81,128,129]. Impaired protein synthesis reduces production of these supportive factors, representing another mechanism through which I1371V astrocytes fail to provide adequate neurotrophic support.

Our findings support a unified model where glucose uptake deficits in LRRK2 I1371V astrocytes trigger organellar dysfunction propagating through the integrated mitochondria–ER–lysosome axis. Reduced glucose uptake and ATP depletion compromise mitochondrial membrane potential maintenance, increasing ROS production. Elevated ROS damages mitochondrial components, impairing respiratory chain function and creating a feed-forward loop of oxidative stress [130]. Simultaneously, ATP depletion compromises lysosomal acidification through impaired v-ATPase function, reducing proteolytic capacity and causing accumulation of damaged organelles and protein aggregates. The ER—deprived of ATP for chaperone function and exposed to oxidative stress from dysfunctional mitochondria—activates the UPR and suppresses protein synthesis, further compromising cellular function.

This integrated dysfunction explains why therapeutic interventions targeting individual organelles have failed to progress or show efficacy in human PD trials [73,74,75,76,77,78]. Restoring glucose metabolism and energy homeostasis represents a more fundamental approach, normalizing function across multiple organellar compartments simultaneously. Metabolic therapies—including ketogenic diets, exercise interventions, and pharmacological metabolic modulators—are attracting increased attention for precisely this reason [131,132].

Our findings identify astrocytic glucose metabolism as a therapeutic target in LRRK2 I1371V-associated PD. Several strategies merit investigation. First, metabolic substrate supplementation with ketone bodies (β-hydroxybutyrate), medium-chain triglycerides, or lactate could bypass glucose uptake deficits and directly fuel oxidative metabolism. Ketogenic diets show promise in preclinical PD models [133,134], and our findings suggest particular benefit for LRRK2 I1371V patients. Second, GLUT1 upregulation via transcriptional regulators such as HIF-1α or improved transporter trafficking could restore glucose uptake capacity [135]. Third, Rab GTPase modulation addresses mechanistic roots: normalizing Rab8A/Rab10 phosphorylation could restore GLUT1 trafficking. LRRK2 kinase inhibitors currently in clinical development [136,137] may partially achieve this, though efficacy in GTPase domain mutations remains to be investigated.

Metabolic interventions offer advantages for early disease-modifying therapy. They are widely accepted and can be initiated in presymptomatic LRRK2 mutation carriers before motor symptoms appear, potentially delaying or preventing disease progression [138,139]. Future work should examine whether glucose metabolism deficits extend across other Roc-COR domain mutations (R1441C/G/H, N1437H, Y1699C) or are specific to I1371V. Longitudinal studies in presymptomatic I1371V carriers identifying when metabolic deficits emerge will define windows for preventive intervention. Developing biomarkers reflecting astrocytic metabolic dysfunction—through PET imaging of glucose metabolism or CSF metabolomic profiling—will enable better patient stratification and therapeutic monitoring.

## 4. Materials and Methods

### 4.1. Reagents

Wild-type (WT) α-synuclein monomeric peptides were procured from rPeptide (Watkinsville, GA, USA), and their endotoxin screening has been documented previously [140]. JC1 and CCCP was purchased from Cayman Chemicals (Ann Arbor, MI, USA). Mitomycin C, dibutyryl cyclic-AMP sodium salt (dbcAMP), the Proteasome 20S Activity Assay Kit, and paraformaldehyde (PFA) were purchased from Sigma-Aldrich (St. Louis, MI, USA). MitoSOX™ Red Mitochondrial Superoxide Indicators, methyl ER-Tracker™ Red (BODIPY™ TR Glibenclamide), Fluo-4 acetoxymethyl (AM), thapsigargin, LysoSensor™ Yellow/Blue DND-160, the Click-iT™ AHA Alexa Fluor™ 488 Protein Synthesis HCS Assay, KnockOut™ Serum Replacement (KOSR), GlutaMAX™, penicillin–streptomycin (penstrep) solution, non-essential amino acid (NEAA) solution, β-mercaptoethanol, DMEM/F12, Geltrex™ matrix, StemFlex™ medium, PSC Neural Induction medium, N-2 Supplement, Advanced DMEM/F12 medium, anti-GLUT1, anti-VDAC1, anti-LAMP1, anti-LAMP2, anti-cathepsin B, anti-cathepsin L, anti-cathepsin D, anti-Mfn2, anti-Fis1, anti-SERCA2b, anti-STIM1, anti-ORAI3, anti- calnexin, anti-IP3R, anti-cytochrome C, anti-phosphorylated PERK, anti-CHOP, anti-Ubiquitin antibodies, dimethyl sulfoxide (DMSO), DAPI, and TRIzol^®^, Mitotracker Green were obtained from Thermo Fisher Scientific (Waltham, MA, USA). Basic fibroblast growth factor (bFGF), epidermal growth factor (EGF), ciliary neurotrophic factor (CNTF), and neuregulin were obtained from ImmunoTools (Friesoythe, Germany). JetPRIME™ DNA transfection reagent was purchased from Polyplus Transfection^®^ (Illkirch, France), and Blasticidin from GBiosciences (St. Louis, MI, USA). Bovine serum albumin (BSA), Triton X-100 and Tween-20, were obtained from Himedia (Maharashtra, India). Cathepsin D Activity Assay Kit (Fluorometric), Alexa Fluor^®^ 488- and Alexa Fluor^®^ 647-conjugated secondary antibodies were purchased from Abcam (Waltham, MA, USA). PrimeScript™ RT Reagent Kit was obtained from Takara (Shiga, Japan), and the SensiFAST™ SYBR^®^ Lo-ROX Kit was purchased from Meridian Bioscience (Newtown, OH, USA). The Cathepsin L Magic Red™ Cathepsin L Kit and the Cathepsin B Green Cathepsin B Kit Rhodamine 110 were purchased from Bio-Rad (Hercules, CA, USA). Glutaraldehyde and Araldite CY212 resin were obtained from TAAB Laboratories Equipment Ltd. (Aldermaston, Berks, England). The details of the antibodies are provided in Supplementary Table S1.

### 4.2. Ethics Clearance

The generation, differentiation, and use of human induced pluripotent stem cells (hiPSCs) were approved by the Institutional Committee for Stem Cell Research (IC-SCR) under approval number SEC/05/030/BP.

### 4.3. Cell Culture

Astrocytes were differentiated from hiPSCs using a two-step protocol optimized in our laboratory [34]. Neural progenitor cells were first exposed to an astrocyte priming medium consisting of Neurobasal medium supplemented with N-2, GlutaMAX™, penicillin–streptomycin, EGF (10 ng/mL), and bFGF (10 ng/mL), thereby generating glial progenitor cells (GPCs) after four days. The cells were subsequently switched to a terminal differentiation medium composed of Neurobasal medium, N-2 supplement, GlutaMAX™, penicillin–streptomycin, CNTF (10 ng/mL), neuregulin-1β (10 ng/mL), and dbcAMP (0.1 µM) for three days, resulting in mature astrocytes. For comparison, the U87 MG glioblastoma cell line (kindly provided by Dr. Nandakumar DN, Department of Neurochemistry, NIMHANS) was cultured in DMEM/F12 supplemented with 10% fetal bovine serum (FBS), GlutaMAX™, and penicillin–streptomycin.

### 4.4. Plasmid Constructs and Transfection

The pDEST51-LRRK2-I1371V (IV) plasmid containing a V5 tag was obtained from Mark Cookson via Addgene (plasmid #29399; RRID:Addgene_29399). An empty vector (EV) control was generated by HindIII digestion of the IV construct to remove the LRRK2 coding sequence (bp 291–6841). Transfections were performed as described previously [34]. Briefly, U87 cells were transfected with EV or IV constructs using JetPRIME™ DNA transfection reagent. Stable transfectants were selected in medium containing 1.5 µg/mL blasticidin. The efficiency of transfection was thoroughly assessed. Expression of LRRK2 at the mRNA level was assessed using both semi-quantitative agarose gel analysis (Supplementary Figure S1A) and quantitative RT-PCR (Supplementary Figure S1B). Agarose gel electrophoresis demonstrated a visibly increased band intensity in I1371V-overexpressing cells compared to empty vector controls, indicating elevated transcript levels. This observation was further validated by qPCR, where fold change was calculated relative to EV U87 cells and normalized to 18S, confirming a statistically significant upregulation of LRRK2 mRNA [*p* < 0.001; EV vs. IV]. Protein expression was verified by Western blot (Supplementary Figure S1C,D; *p* < 0.001 EV vs. IV), revealing a distinct band with markedly higher intensity in IV-transfected cells. Additionally, transfection efficiency was evaluated by measuring the population of LRRK2-positive cells using flow cytometry, which showed significantly higher expression of LRRK2 in IV cells in comparison to EV cells, which is similar to the expression pattern in HC and PD astrocytes (Supplementary Figure S1E–H; *p* < 0.001 HC vs. PD; EV vs. IV).

### 4.5. Immunocytochemistry (ICC)

Adherent cells seeded on 12 mm coverslips at 90–95% confluency were fixed with 4% PFA. Permeabilization was performed using 1% Triton X-100, followed by blocking with 3% BSA. Primary antibodies (anti-GLUT1, anti-Ubiquitin, anti-VDAC1, anti-Mfn2, anti-Fis1, anti- SERCA2b, anti-calnexin, anti-IP3R, anti-cytochrome C, anti-LAMP1, anti-LAMP2, anti-cathepsin B, anti-cathepsin L, and anti-cathepsin D) were used at 1:100 dilution. Permeabilization was not performed for cell surface marker expression (STIM1 and ORAI3). Alexa Fluor^®^ 488- and 647-tagged secondary antibodies were used at 1:200 dilution. Nuclei were stained with 300 nM DAPI. Washes were performed in PBS with 0.05% Tween-20. Confocal microscopy was carried out using a Zeiss LSM 980 with Airyscan super-resolution (Axio Observer.Z1/7) with a Plan Apochromat 40×/1.3 Oil DIC (UV) VIS-IR M27 objective (Carl Zeiss, Oberkochen, Germany). At least three biological replicates were analyzed with ≥10 fields imaged per condition. Image acquisition and analysis were performed using ZEISS ZEN 3.7 software, including generation of 2.5D views and calculation of Pearson’s correlation coefficients (n ≥ 5). Secondary antibody-only controls (no primary antibody) were processed in parallel to assess non-specific background, confirming that the observed fluorescence signals were primary antibody-dependent; representative images are provided in Supplementary Figure S2A,B.

### 4.6. Flow Cytometry

Astrocytes and U87 transfected cells were dissociated with StemPro™ Accutase™. Cells were fixed in 2% PFA for 45 min at room temperature, centrifuged at 10,000 rpm for 10 min (4 °C), and resuspended in DPBS with 0.01% sodium azide (1 × 10^5^ cells/reaction). For intracellular markers (GLUT1, Ubiquitin, VDAC1, Mfn2, Fis1, SERCA2b, calnexin, IP3R, cytochrome C, LAMP1, LAMP2, cathepsin B, cathepsin L, cathepsin D, p-PERK, CHOP), permeabilization was performed with 1% Triton X-100 for 15 min. Permeabilization was not performed for cell surface marker expression (STIM1 and ORAI3). Cells were blocked in 3% BSA for 45 min and incubated with primary antibodies (16 h, 4 °C), followed by fluorophore-tagged secondary antibodies (90 min, RT). For the surface expression of GLUT1, permeabilization step was omitted. Acquisition was performed on a BD FACSLyric flow cytometer with 10,000 events/sample. FSC versus SSC scatter plots were used to define the intact cell population without selective exclusion (Supplementary Figure S2C–F), and isotype controls for Alexa Fluor 488 and 647 were included to establish non-specific fluorescence thresholds; representative FSC/SSC plots and isotype controls are provided in Supplementary Figure S2G–N.

### 4.7. Quantitative Polymerase Chain Reaction (qPCR)

As described in our previous publications [34,141], qPCR was performed and analyzed using cDNA from treated and untreated HC and PD astrocytes. The primers used were 18S (Forward: 5′-CGGCTACCACATCCAAGGAA-3′, Reverse: 5′-GCTGGAATTACCGCGGCT-3′), GADD34 (Forward: 5′-TCCGACTGCAAAGGCGGCTCA-3′, Reverse: 5′-CAGCCAGGAAATGGACAGTGAC-3′), CHOP (Forward: 5′-GGTATGAGGACCTGCAAGAGGT-3′, Reverse: 5′-CTTGTGACCTCTGCTGGTTCTG-3′), and LRRK2 (Forward: 5′- ATGAGATATGCACTCTTCTG-3′, Reverse: 5′-GCAACCGATCAAAGTACCTAGC-3′). The qPCR was conducted using the CFX Opus 96 Real-Time PCR System (Bio Rad Laboratories), and data analysis was done with CFX Maestro 2.3 software. Results were represented as fold change in mRNA levels in PD and treated astrocytes, normalized to 18S mRNA and iNOS mRNA levels in healthy control (HC) astrocytes.

### 4.8. Glucose Uptake Assay

Glucose uptake in HC and PD astrocytes was evaluated using the fluorescent D-glucose analogue 2-(N-(7-Nitrobenz-2-oxa-1,3-diazol-4-yl)Amino)-2-Deoxyglucose (2-NBDG; TCI). HC and PD astrocytes cultured on Geltrex coated plates were washed with PBS to remove residual glucose before incubation. The cells were then exposed to 50 μM 2-NBDG in HBSS (without glucose) at 37 °C for 30 min. After incubation, they were washed twice with cold PBS, detached using Accutase, and centrifuged. The resulting cell pellet was resuspended in PBS, and fluorescence intensity was measured using a BD FACSLyric flow cytometer. Mean fluorescence intensity was compared between healthy control (HC) and Parkinson’s disease (PD) astrocytes to assess relative glucose uptake efficiency.

### 4.9. Lactate Production Assay

Lactate levels in the conditioned media were quantified using the L-Lactate Assay Kit (Elabscience, Houston, TX, USA) according to the manufacturer’s instructions. Absorbance was measured at 530 nm using an Infinite^®^ 200 microplate reader (Tecan, Männedorf, Switzerland) to determine lactate release.

### 4.10. Mitochondrial Membrane Potential

The mitochondrial membrane potential was determined in the control and peptide-treated cells (24 and 48 h) using the lipophilic cationic dye 5′,6,6′-tetrachloro-1,1′,3,3′-tetraethylbenzimidazolylcarbocyanine iodide (JC-1) as per the manufacturer’s instructions [80,81]. Dual analysis in the form of a bivariate plot was measured using FACS Lyric ((BD Biosciences, Franklin Lakes, NJ, USA)and analyzed using the software BD FACSuite Software Version 1.4. Additionally, red-to-green emission intensity ratio was plotted in the form of a graph.

### 4.11. Mitochondrial Superoxide Assay

Cells seeded on 12 mm coverslips at 90–95% confluency were stained with MitoSOX™ Red (Thermo Fisher Scientific (Waltham, MA, USA)) according to the manufacturer’s instructions. After staining, cells were imaged on a Zeiss LSM 980 confocal microscope (Axio Observer.Z1/7) (Carl Zeiss, Oberkochen, Germany) using the same optical configuration described above. Three biological replicates were analyzed, with at least ten fields captured per sample. Quantitative analysis was carried out in ImageJ (RRID:SCR_003070), and CTCF was calculated from 50 ROIs using the same formula employed for ER-Tracker fluorescence [142,143]. 

### 4.12. Mitotracker Green Staining

Mitochondrial mass was assessed using MitoTracker Green (MTG) [Thermo Fisher Scientific (Waltham, MA, USA)], which labels mitochondria in a membrane potential-independent manner. Cells were incubated with 300 nM MTG and 300 nM DAPI, followed by confocal microscopy imaging.

### 4.13. 20S Proteasome Activity Assay

Proteasome activity was assessed in 80,000 control and α-synuclein monomer-treated astrocytes seeded per well in a 96-well plate using the Proteasome 20S Activity Assay Kit, following the manufacturer’s instructions. Fluorescence intensity was measured at an excitation wavelength of 490 nm and an emission wavelength of 525 nm using an Infinite^®^ 200 microplate reader (Tecan).

### 4.14. Intracellular Cathepsin Activity Assays

Intracellular cathepsin activities were measured using the Magic Red™ Cathepsin L Detection Kit, the Green cathepsin B Detection Kit, and the Cathepsin D Activity Assay Kit, following the manufacturers’ instructions. Cathepsin L and D activities were assessed using a fluorometric format on an Infinite^®^ 200 microplate reader (Tecan) with excitation/emission wavelengths of 592/628 nm for Cathepsin L and 328/460 nm for Cathepsin D. Cathepsin B activity was measured in live cells using flow cytometry with an excitation/emission of 488/525 nm. All measurements were performed five times, and data were used to quantify enzyme activity in HC and PD astrocytes.

### 4.15. Lysosomal pH Measurement

Lysosomal pH was determined using LysoSensor™ Yellow/Blue DND-160 (Thermo Fisher Scientific). Cells grown on coverslips at 90–95% confluency were stained with 1 µM probe in prewarmed growth medium for 5 min at 37 °C, as per manufacturer’s instructions. After incubation, the dye was replaced with fresh medium, and coverslips were mounted directly for imaging. Fluorescence was acquired on a Zeiss LSM 980 confocal microscope using the Plan Apochromat 40×/1.3 Oil DIC objective. For each condition, five independent samples were processed with ≥10 fields per replicate. Quantitative analysis of fluorescence was performed in ImageJ using CTCF from 50 ROIs as described earlier [142,143].

### 4.16. Transmission Electron Microscopy (TEM)

Cells from at least two 60 mm dishes (~95% confluency) were processed for TEM as described previously [84,87]. Cells were primarily fixed in 3% buffered glutaraldehyde for 1 h, post fixed 1% osmium tetroxide for 1 h. Sodium cacodylate buffer (0.1 M, pH-7.2–7.4) was used for washes between fixation steps. Samples were then dehydrated through a graded ethanol series, cleared in propylene oxide, and embedded in Araldite CY212 resin, which was polymerized at 60 °C for 48 h. Ultrathin sections were cut using a Leica EM UC7 ultramicrotome (Leica Mikrosysteme, Vienna, Austria) and contrasted with saturated methnolic uranyl acetate and lead citrate. Imaging was performed on a JEM-1400 Plus TEM (JEOL Ltd., Tokyo, Japan) operated at 80 kV, with images captured using a Gatan SC1000B camera (Gatan, Inc., Pleasanton, CA, USA) [144].

### 4.17. Endoplasmic Reticulum (ER)-Tracker™ Labelling and Imaging

Cells grown on 12 mm coverslips at ~90–95% confluency were used for ER staining. All washes were performed with Hank’s balanced salt solution (HBSS; pH 7.4). Cells were incubated with 1 µM ER-Tracker™ Red following the manufacturer’s protocol and subsequently fixed with 4% paraformaldehyde. Imaging was performed on a Zeiss LSM 980 confocal microscope (Axio Observer.Z1/7) equipped with a Plan Apochromat 40×/1.3 Oil DIC (UV) VIS-IR M27 objective (Carl Zeiss, Oberkochen, Germany). For each condition, three independent samples were processed and a minimum of ten fields were acquired. Images were analyzed using ImageJ software (RRID:SCR_003070), and corrected total cell fluorescence (CTCF) was calculated from 50 regions of interest (ROIs) using the formula: CTCF = Integrated density − (Area of selected cell × Mean background fluorescence), as described previously [142,143].

### 4.18. ER Calcium Dynamics

Adherent cells grown on 22 mm coverslips at 90–95% confluency were used for live-cell fluorescence assays. Intracellular calcium [Ca^2+^]i was measured using the cell permeant fluorescent indicator Fluo-4 AM, following protocols described previously [86,88]. The coverslip was mounted in a perfusion chamber (bath volume: 400 µL) placed on the stage of a Leica DMi8 inverted fluorescence microscope. Stained cells were imaged with a Semi-Apochromats 40×/0.80 PL FLUOTAR objective, connected to a Leica DFC3000G camera, and operated via LAS X software software version 3.3.3.16958 (Leica Application Suite X, RRID:SCR_013673). Time-lapse imaging was performed at 500 ms intervals for 201 s to record fluorescence intensity.

To specifically assess calcium entry through calcium release-activated calcium (CRAC) channels, cells were perfused with calcium-free buffer supplemented with EGTA. Store-operated calcium entry (SOCE) was induced by inhibiting the endoplasmic reticulum calcium ATPase (SERCA) pump with 10 µM thapsigargin for 10 min, followed by extracellular addition of 2 mM CaCl_2_. Fluorescence changes during stimulation were recorded under identical conditions. Data were analyzed using ImageJ software (RRID:SCR_003070), and imaging results were expressed as F1/F0 ratios. For quantitative comparison, the area under the curve (AUC) was calculated for each experimental group.

### 4.19. Measurement of Nascent Protein Synthesis Using Click-iT™ HPG Assay

Nascent protein synthesis was assessed using the Click-iT™ HPG Alexa Fluor™ 488 Protein Synthesis Assay Kit (Thermo Fisher Scientific) according to the manufacturer’s instructions. Following completion of the reaction, cells were washed thoroughly with reaction rinse buffer and nuclei were counterstained with DAPI. Imaging was performed on a Zeiss LSM 980 confocal microscope (Axio Observer.Z1/7) equipped with a Plan Apochromat 40×/1.3 Oil DIC (UV) VIS-IR M27 objective. For each condition, three independent samples were processed and a minimum of ten fields were acquired. Images were analyzed using ImageJ software (RRID:SCR_003070), and corrected total cell fluorescence (CTCF) was calculated from 50 regions of interest (ROIs) using the formula: CTCF = Integrated density − (Area of selected cell × Mean background fluorescence).

### 4.20. Immunoblotting

Protein lysates were obtained from cultured cells, and 40 μg of total protein was loaded per lane in accordance with previously established protocols [34]. Samples were resolved by SDS–PAGE and subsequently transferred onto PVDF membranes using a semidry transfer system, with a pre-stained protein ladder. Membranes were blocked with 5% BSA and incubated with primary antibodies (1:1000 dilution) targeting LRRK2 and β-actin. Detection was carried out using fluorescently labelled secondary antibodies, with anti-mouse for β-actin and anti-rabbit for LRRK2 applied at a 1:10,000 dilution. Between each step, membranes were washed with TBST (0.1% Tween-20). Protein bands were visualized using a fluorescence imaging system, and densitometric analysis was performed using ImageJ (v1.52f), with LRRK2 expression normalized to β-actin and represented as relative density.

### 4.21. Statistics

All results are expressed as mean ± SD. Statistical comparisons were performed using two-sample *t*-test or one-way ANOVA, as appropriate, followed by Bonferroni post hoc analysis, using R software version 3.4.1 (R Foundation; R Project for Statistical Computing). A *p*-value less than 0.05 was considered significant. Graphs were created using GraphPad Prism 8 (GraphPad Software) or Sigma Plot 12.5. Unless otherwise stated, graphed data are presented as means ± SD. Single symbol: *p* < 0.05; double-symbol: *p* < 0.01 triple-symbol: *p* < 0.001.

## 5. Conclusions

The LRRK2 I1371V mutation disrupts astrocytic energy homeostasis through several interconnected mechanisms. Reduced GLUT1-mediated glucose uptake limits glycolysis and decreases neuronal lactate supply. Mitochondrial dysfunction marked by depolarization and elevated ROS amplifies energy stress. Compromised proteasomal activity and lysosomal deficits—including reduced LAMP1/LAMP2, impaired acidification, and selective cathepsin D loss—hinder clearance of damaged organelles and α-synuclein. ER stress and UPR activation suppress protein synthesis, reducing expression of critical transporters and enzymes including GLUT1 and SLC1A2. These deficits create self-reinforcing cycles where energy depletion, impaired proteostasis, and organellar dysfunction perpetuate cellular failure. Our findings identify astrocytic metabolic capacity as a therapeutic target, suggesting that interventions restoring energy homeostasis and support functions—particularly when initiated early—could provide disease-modifying benefits in LRRK2 I1371V-associated PD.

## Data Availability

All data generated or analyzed during this study are included in this published article and its supplementary information files.

## Supplementary Materials

The following supporting information can be downloaded at: https://www.mdpi.com/article/10.3390/ijms27083463/s1.

## Abbreviations

The following abbreviations are used in this manuscript:

PD	Parkinson’s Disease
iPSC	induced Pluripotent Stem Cell
HC	Healthy Control
LRRK2	Leucine-rich Repeat Kinase 2
GLUT1	Glucose Transporter 1
LAMP1	Lysosome-Associated Membrane Protein 1
LAMP2	Lysosome-Associated Membrane Protein 2
GADD34	Growth Arrest and DNA Damage-Inducible Protein 34
CHOP	C/EBP Homologous Protein
STIM1	Stromal Interaction Molecule 1
ORAI3	ORAI Calcium Release-Activated Calcium Modulator 3
MAMs	Mitochondria-Associated Membranes
PERK	PKR-like Endoplasmic Reticulum Kinase
Roc	Ras of Complex Proteins
COR	C-terminal of Roc
ANLS	Astrocyte–Neuron Lactate Shuttle
ATP	Adenosine Triphosphate
ER	Endoplasmic Reticulum
MCT4	Monocarboxylate Transporter 4 (SLC16A3)
2-NBDG	2-(N-(7-Nitrobenz-2-oxa-1,3-diazol-4-yl)amino)-2-Deoxy-D-Glucose
JC-1	5,5′,6,6′-Tetrachloro-1,1′,3,3′-Tetraethylbenzimidazolylcarbocyanine Iodide
CCCP	Carbonyl Cyanide m-Chlorophenyl Hydrazone
EV	Cells transfected with Empty Vector plasmid
IV	Cells transfected with LRRK2 I1371V mutation plasmid
Ubq	Ubiquitin
VDAC1	Voltage-Dependent Anion Channel 1
CTCF	Corrected Total Cell Fluorescence
ROS	Reactive Oxygen Species
FACS	Fluorescence Activated Cell Sorting
MFN2	Mitofusin 2
TEM	Transmission Electron Microscopy
SERCA2	Sarco/Endoplasmic Reticulum Ca^2+^-ATPase 2
SOCE	Store-Operated Calcium Entry
GBA1	Glucocerebrosidase 1
TFEB	Transcription Factor EB
GDNF	Glial Cell Line Derived Neurotrophic Factor
BDNF	Brain-Derived Neurotrophic Factor
HIF-1α	Hypoxia Inducible Factor 1 Alpha
DNA	Deoxyribonucleic Acid
PCR	Polymerase Chain Reaction
mRNA	Messenger Ribonucleic Acid
SD	Standard Deviation
NP	Neural Progenitors
EGF	Epidermal Growth Factor
bFGF	Basic Fibroblast Growth Factor
GPC	Glial Progenitor Cells
CNTF	Ciliary Neurotrophic Factor
BSA	Bovine Serum Albumin
ICC	Immunocytochemistry
cDNA	Complementary DNA
