# Supplementary material for: LRRK2 I1371V Mutation Drives Astrocytic Glucose Metabolism Failure and Induces Integrated ER–Mitochondria–Lysosome Dysfunction in Parkinson’s Disease
Source: Int J Mol Sci. 2026 Apr 12;27(8):3463. doi: 10.3390/ijms27083463 (PMC13116896; doi:10.3390/ijms27083463)
Supplement: Supplementary file 1 [file ijms-27-03463-s001.zip › ijms-4205655-supplementary.pdf]

Supplementary Table S1: Research Resource Identifiers (RRID) and other details of the antibodies used in the study:

S. No.	Name (target)	RRID citation	Species raised in	Concentration used	Link to website
1	Anti-GLUT1 (GLUT1)	(Thermo Fisher Scientific Cat# MA5-31960, RRID:AB_2809254)	Rabbit	1:100 (Immunocyto-chemistry and Flow cytometry)	https://www.thermofisher.com/antibody/product/GLUT1-Antibody-clone-SA0377-Recombinant-Monoclonal/MA5-31960
2	Anti-VDAC1 (VDAC1)	(Thermo Fisher Scientific Cat# 433110, RRID: AB_3731326)	Mouse	1:100 (Immunocyto-chemistry and Flow cytometry)	https://www.thermofisher.com/antibody/product/VDAC1-Antibody-clone-S152B-23-Monoclonal/56564-100UG
3	Anti-LAMP1 (LAMP1)	(Thermo Fisher Scientific Cat# 14-1079-80, RRID:AB_467426)	Mouse	1:100 (Immunocyto-chemistry and Flow cytometry)	https://www.thermofisher.com/antibody/product/CD107a-LAMP-1-Antibody-clone-eBioH4A3-Monoclonal/14-1079-80
4	Anti-LAMP2 (LAMP2)	(Thermo Fisher Scientific Cat# PA1-655, RRID:AB_2134625)	Rabbit	1:100 (Immunocyto-chemistry and Flow cytometry)	https://www.thermofisher.com/antibody/product/LAMP2-Antibody-Polyclonal/PA1-655

5	Anti-Cathepsin B (Cathepsin B)	(Thermo Fisher Scientific Cat# PA5-82618, RRID:AB_2789775)	Rabbit	1:100 (Immunocyto-chemistry and Flow cytometry)	https://www.thermofisher.com/antibody/product/Cathepsin-B-Antibody-Polyclonal/PA5-82618
6	Anti-Cathepsin L (Cathepsin L)	(Thermo Fisher Scientific Cat# BMS1032, RRID:AB_10596643)	Mouse	1:100 (Immunocyto-chemistry and Flow cytometry)	https://www.thermofisher.com/antibody/product/Cathepsin-L-Antibody-clone-33-2-Monoclonal/BMS1032
7	Anti-Cathepsin D (Cathepsin D)	(Thermo Fisher Scientific Cat# MA5-32127, RRID:AB_2809418)	Rabbit	1:100 (Immunocyto-chemistry andFlow cytometry)	https://www.thermofisher.com/antibody/product/Cathepsin-D-Antibody-clone-SU0360-Recombinant-Monoclonal/MA5-32127
8	Anti-MFN2 (MFN2)	(Thermo Fisher Scientific Cat# PA5-118059, RRID:AB_2902662)	Rabbit	1:100 (Immunocyto-chemistry and Flow cytometry)	https://www.thermofisher.com/antibody/product/MFN2-Antibody-Polyclonal/PA5-118059
9	Anti-FIS1 (FIS1)	(Thermo Fisher Scientific Cat# PA5-22142, RRID:AB_11152577)	Rabbit	1:100 (Immunocyto-chemistry and Flow cytometry)	https://www.thermofisher.com/antibody/product/FIS1-Antibody-Polyclonal/PA5-22142

10	Anti-SERCA2 (SERCA2)	(Thermo Fisher Scientific Cat# MA3-919, RRID:AB_325502)	Mouse	1:100 (Immunocyto-chemistry and Flow cytometry)	https://www.thermofisher.com/antibody/product/SERCA2-ATPase-Antibody-clone-2A7-A1-Monoclonal/MA3-919
11	Anti-STIM1 (STIM1)	(Thermo Fisher Scientific Cat# PA5-82455, RRID:AB_2789613)	Rabbit	1:100 (Immunocyto-chemistry and flow cytometry)	https://www.thermofisher.com/antibody/product/STIM1-Antibody-Polyclonal/PA5-82455
12	Anti-ORAI3 (ORAI3)	(Thermo Fisher Scientific Cat# MA5-15778, RRID:AB_11156221)	Mouse	1:100 (Immunocyto-chemistry and flow cytometry)	https://www.thermofisher.com/antibody/product/ORAI3-Antibody-clone-2H2G9-Monoclonal/MA5-15778
13	Anti-Calnexin (Calnexin)	(Thermo Fisher Scientific Cat# MA3-027, RRID:AB_2069043)	Mouse	1:100 (Immunocyto-chemistry and flow cytometry)	https://www.thermofisher.com/antibody/product/Calnexin-Antibody-clone-AF18-Monoclonal/MA3-027
14	Anti-IP3R (IP3R)	(Thermo Fisher Scientific Cat# PA1-901, RRID:AB_2129984)	Rabbit	1:100 (Immunocyto-chemistry and flow cytometry)	https://www.thermofisher.com/antibody/product/IP3-Receptor-1-Antibody-

					Polyclonal/PA1-901
15	Anti-Cytochrome C (Cyt C)	(Thermo Fisher Scientific Cat# MA5-11674, RRID:AB_10985701)	Mouse	1:100 (Immunocyto-chemistry and flow cytometry)	https://www.thermofisher.com/antibody/product/Cytochrome-C-Antibody-clone-7H8-2C12-Monoclonal/MA5-11674
16	Anti-phosphorylated PERK (p-PERK)	Bioss Cat# bs-3330R, RRID:AB_10855345	Rabbit	1:100 (flow cytometry)	https://www.biossusa.com/products/bs-3330r
17	Anti-CHOP (p-CHOP)	(Thermo Fisher Scientific Cat# PA5-104528, RRID:AB_2853828)	Rabbit	1:100 (flow cytometry)	https://www.thermofisher.com/antibody/product/CHOP-Antibody-Polyclonal/PA5-104528
18	Anti-Ubiquitin (Ubiquitin)	Thermo Fisher Scientific Cat# PA5-32634, RRID:AB_2550097	Rabbit	1:100 (Immunocyto-chemistry and flow cytometry)	https://www.thermofisher.com/antibody/primary/PA5-32634
19	Secondary antibody tagged with Alexa Fluor® 488 (IgG H&L)	Abcam Cat# ab150113, RRID:AB_2576208<br><br>Abcam Cat# ab150077, RRID:AB_2630356	Goat against mouse<br><br>Goat against rabbit	1:200 (Immunocyto-chemistry and flow cytometry)	https://www.abcam.com/goat-mouse-igg-hl-alexa-fluor-488-ab150113.html<br><br>abcam.com/goat-rabbit-igg-hl-alexa-fluor-488-ab150077.html

20	Secondary antibody tagged with Alexa Fluor® 647 (IgG H&L)	Abcam Cat# ab150115, RRID:AB_2687948<br><br>Abcam Cat# ab150075, RRID:AB_2752244	Goat against mouse<br><br>Donkey against rabbit	1:200 (Immunocyto-chemistry and flow cytometry)	https://www.abcam.com/goat-mouse-igg-hl-alexa-fluor-647-ab150115.html<br><br>https://www.abcam.com/donkey-rabbit-igg-hl-alexa-fluor-647-ab150075.html

Supplementary figures:

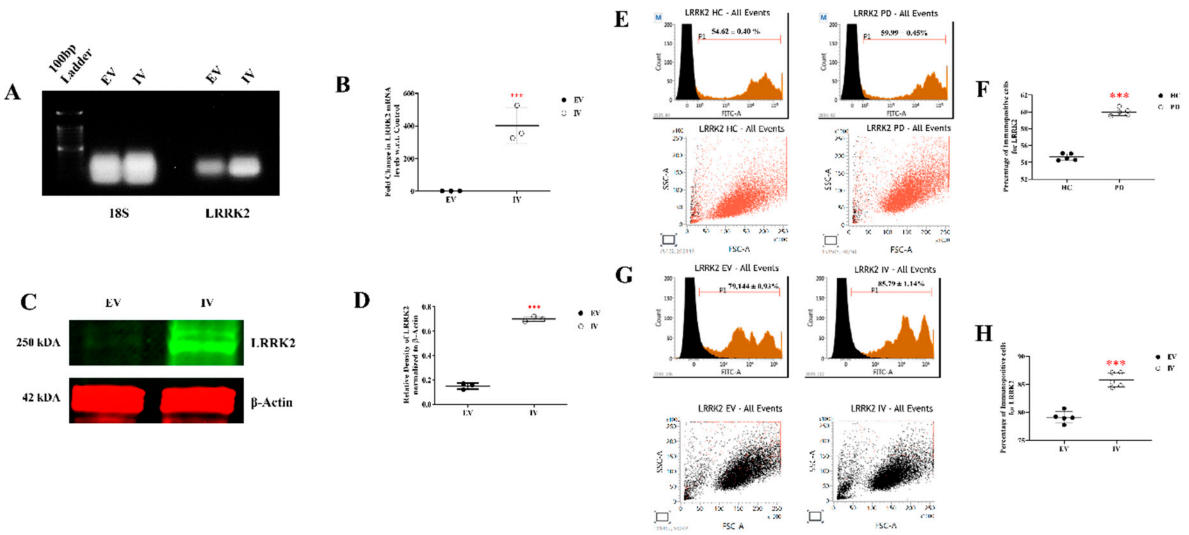

Figure S1. (A) Semi-quantitative RT-PCR analysis of LRRK2 mRNA expression in transfected U87 cells. Representative agarose gel image is shown. Lane 1: DNA ladder; Lanes 2–3 18S rRNA housekeeping gene (187 bp amplicon) in EV and IV respectively; and in Lanes 5-6 : LRRK2 mRNA (573 bp amplicon) in EV and IV cells, respectively. **(B)** Quantification of mRNA gene expression of LRRK2 in EV and IV transfected U87 cells using quantitative polymerase chain reaction (qPCR) [$p<0.001$; EV vs IV] (Data are mean ± SD, Two Sample t-Test; n=3). **(C)** Western blot bands obtained using antibodies against LRRK2 from 40µg of extracted cell lysate in EV and IV transfected U87 cells. **(D)** Densitometry plot of the bands obtained in western blot. Increased protein levels detected with IV cells [$p < 0.001$; EV vs IV], (Data are mean ± SD, Two Sample t-Test; n=3). **(E)** Representative FACs Histogram and Scatter plots of LRRK2 expression in HC and PD Astrocytes. **(F)** Flow Cytometric Analysis of LRRK2 expression in HC and PD astrocytes. [$p<0.001$ HC vs PD] ((Data are mean ± SD,

Two Sample t-Test; n=5). **(G)** Representative FACs Histogram and Scatter plots of LRRK2 expression in EV and IV transfected U87 Cells. **(H)** Flow Cytometric Analysis of LRRK2 expression in EV and IV transfected U87 Cells [p<0.001 EV vs IV] ((Data are mean ± SD, Two Sample t-Test; n=5).

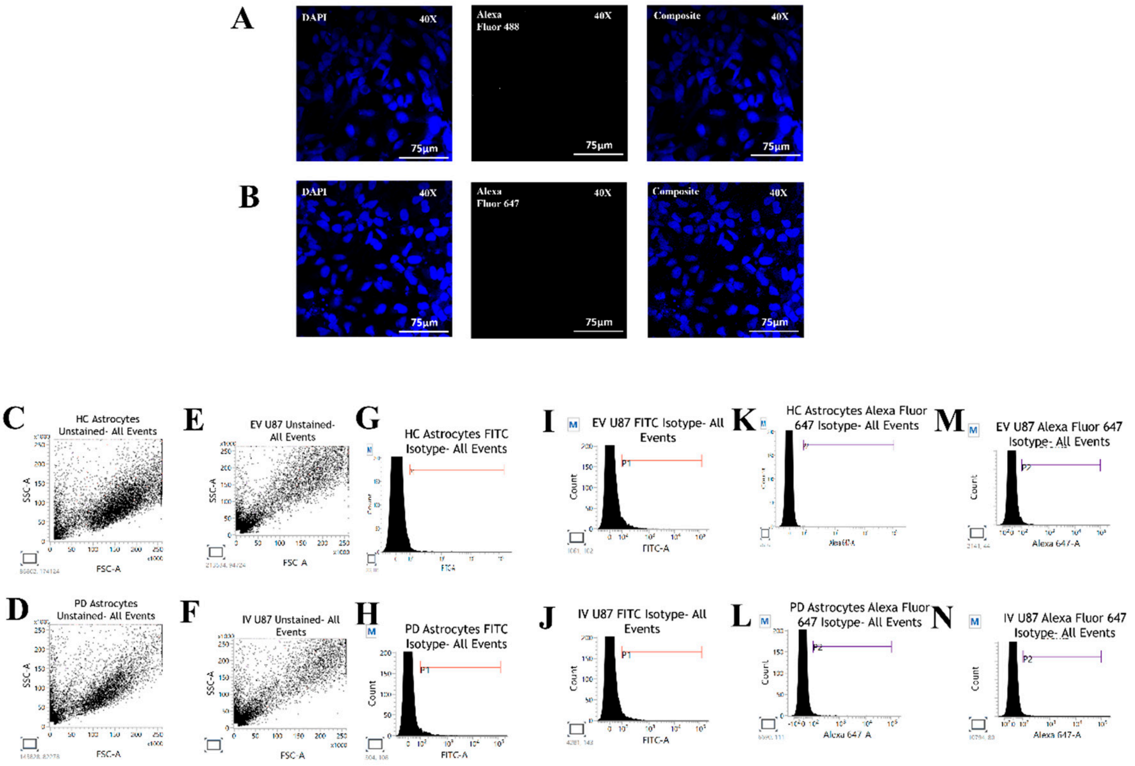

Figure S2. (A-B) Representative ICC images of Astrocytes stained with secondary antibodies tagged with Alexa Fluor 488 **(A)** and Alexa Fluor 647 **(B)** applied in the absence of any primary antibody. **(C-F)** Representative Scatter plots of HC (C), PD (D), EV (E) and IV (F) cells stained with Secondary Antibody in the absence of Primary Antibody. **(G-J)** Representative Flow Histograms of HC (G), PD (H), EV (I) and IV (J) cells stained with Secondary Antibody tagged with Alexa Fluor 488 in the absence of Primary Antibody. Representative Flow Histograms of HC **(K)**, PD **(L)**, EV **(M)** and IV **(N)** cells stained with Secondary Antibody tagged with Alexa Fluor 647 in the absence of Primary Antibody.

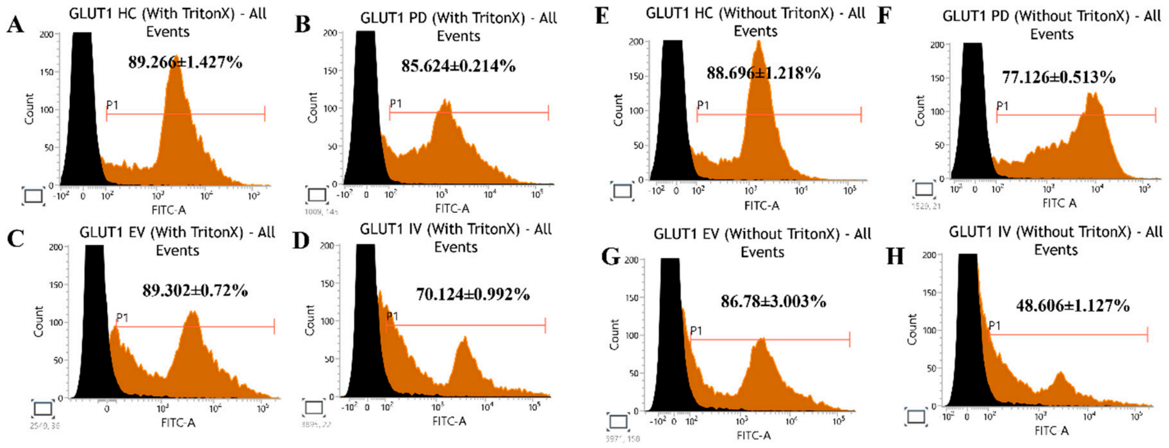

Figure S3. (A-B) Representative flow cytometry histogram of total GLUT1 in HC **(A)** and PD **(B)** astrocytes. **(C-D)** Representative flow cytometry histogram of total GLUT1 in EV **(C)** and IV **(D)** transfected U87 cells. **(E-F)** Representative flow cytometry histogram of membrane localized GLUT1 in HC **(E)** and PD **(F)** astrocytes. **(G-H)** Representative flow cytometry histogram of total GLUT1 in EV **(G)** and IV **(H)** transfected U87 cells.

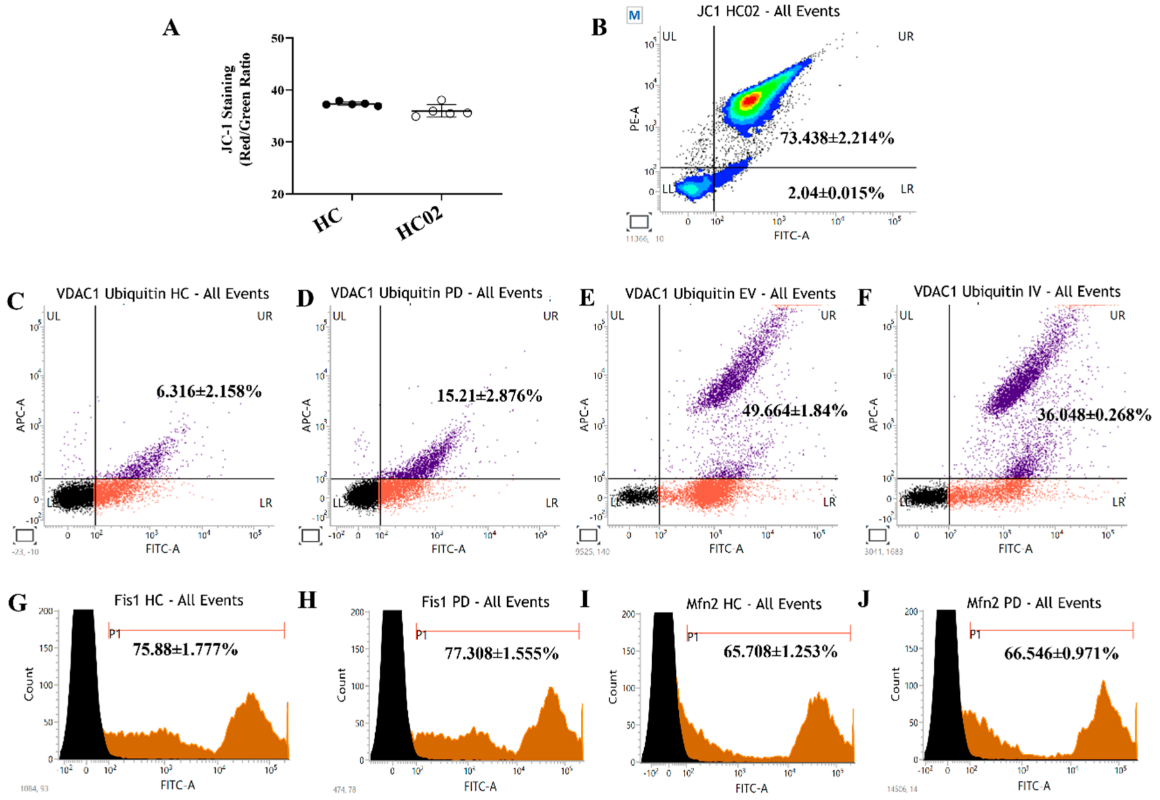

Figure S4. (A) Quantification of JC-1 aggregate/monomer ratios in HC and HC02 Astrocytes. (n=5) **(B)** Representative JC-1 flow cytometry histogram illustrating red (aggregate) to green (monomer) fluorescence in HC02 astrocytes. **(C-D)** Representative Scatter Plot of dual positive VDAC1 and Ubiquitin population of cells in HC **(C)** and PD **(D)** astrocytes. **(E-F)** Representative Scatter Plot of dual positive VDAC1 and Ubiquitin population of cells in EV **(E)** and IV **(F)** transfected U87 cells. **(G-H)** Representative flow cytometry histogram of Fis1 in HC **(G)** and PD **(H)** astrocytes. **(I-J)** Representative flow cytometry histogram of Mfn2 in HC **(I)** and PD **(J)** astrocytes.

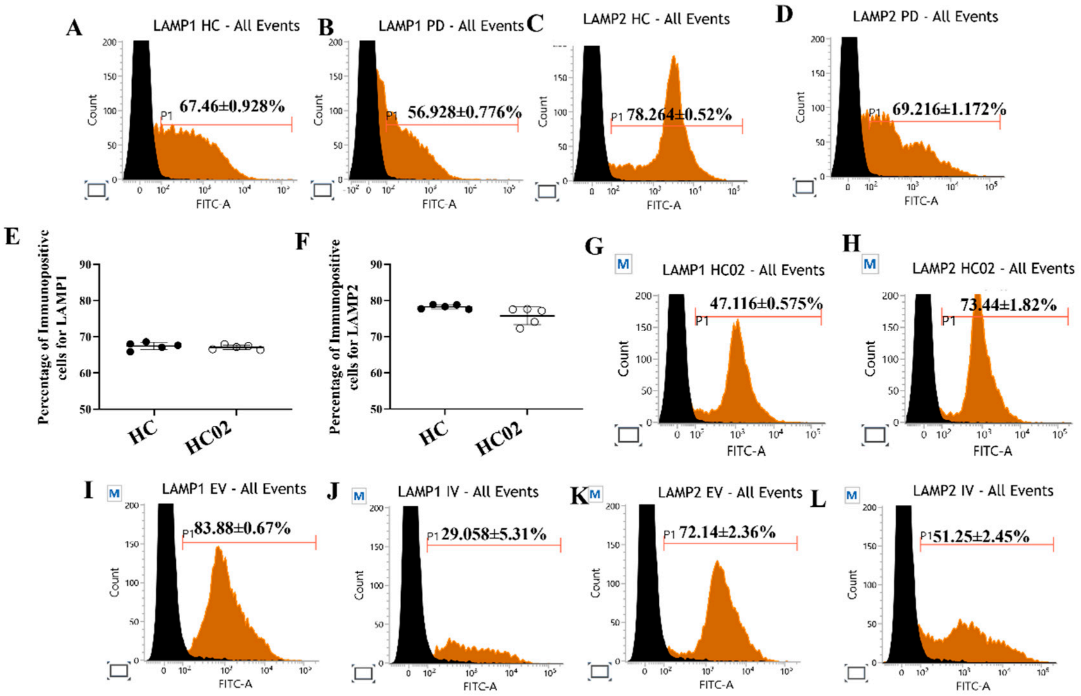

Figure S5. (A-B) Representative flow cytometry histogram of LAMP1 in HC **(A)** and PD **(B)** astrocytes. **(C-D)** Representative flow cytometry histogram of LAMP2 in HC **(C)** and PD **(D)** astrocytes. **(E)** Graphical representation of the flow cytometry quantified positive population for LAMP1 in HC and HC02 astrocytes. (n=5) **(F)** Graphical representation of the flow cytometry quantified positive population for LAMP2 in HC and HC02 astrocytes. (n=5) **(G)** Representative flow cytometry histogram of LAMP1 in HC02 astrocytes. **(H)** Representative flow cytometry histogram of LAMP2 in HC02 astrocytes. **(I-J)** Representative flow cytometry histogram of LAMP1 in EV **(I)** and IV **(J)** transfected U87 cells. **(K-L)** Representative flow cytometry histogram of LAMP2 in EV **(K)** and IV **(L)** transfected U87 cells.

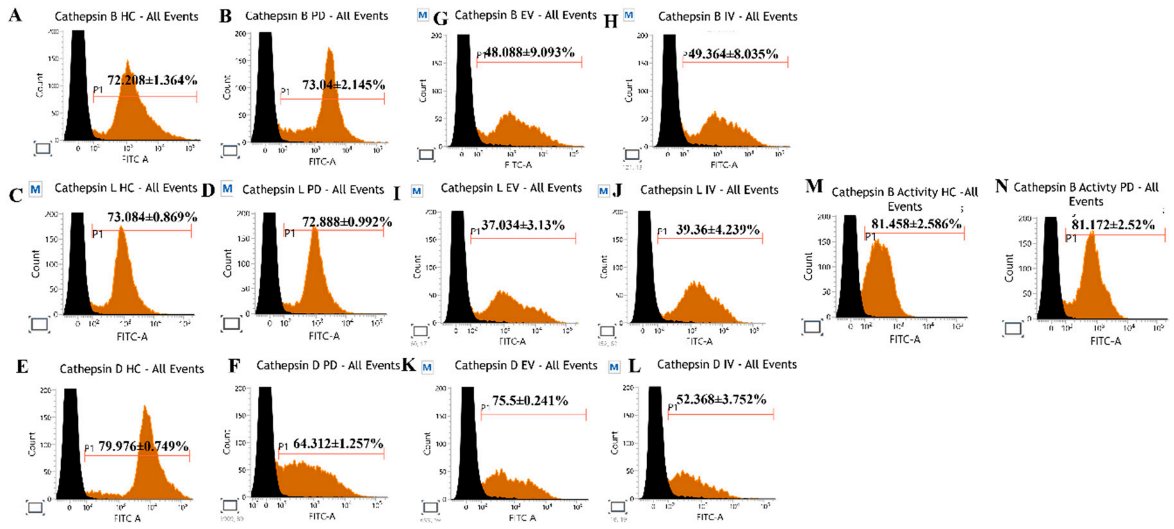

Figure S6. Flow Cytometry Histograms. (A-B) Representative flow cytometry histogram of Cathepsin B in HC **(A)** and PD **(B)** astrocytes. **(C-D)** Representative flow cytometry histogram of Cathepsin L in HC **(C)** and PD **(D)** astrocytes. **(E-F)** Representative flow

cytometry histogram of Cathepsin D in HC **(E)** and PD **(F)** astrocytes. **(G-H)** Representative flow cytometry histogram of Cathepsin B in EV **(G)** and IV **(H)** transfected U87 cells. **(I-J)** Representative flow cytometry histogram of Cathepsin L in EV **(I)** and IV **(J)** transfected U87 cells. **(K-L)** Representative flow cytometry histogram of Cathepsin D in EV **(K)** and IV **(L)** transfected U87 cells. **(M-N)** Representative flow cytometry histogram of Cathepsin D in HC **(M)** and PD **(N)** astrocytes.

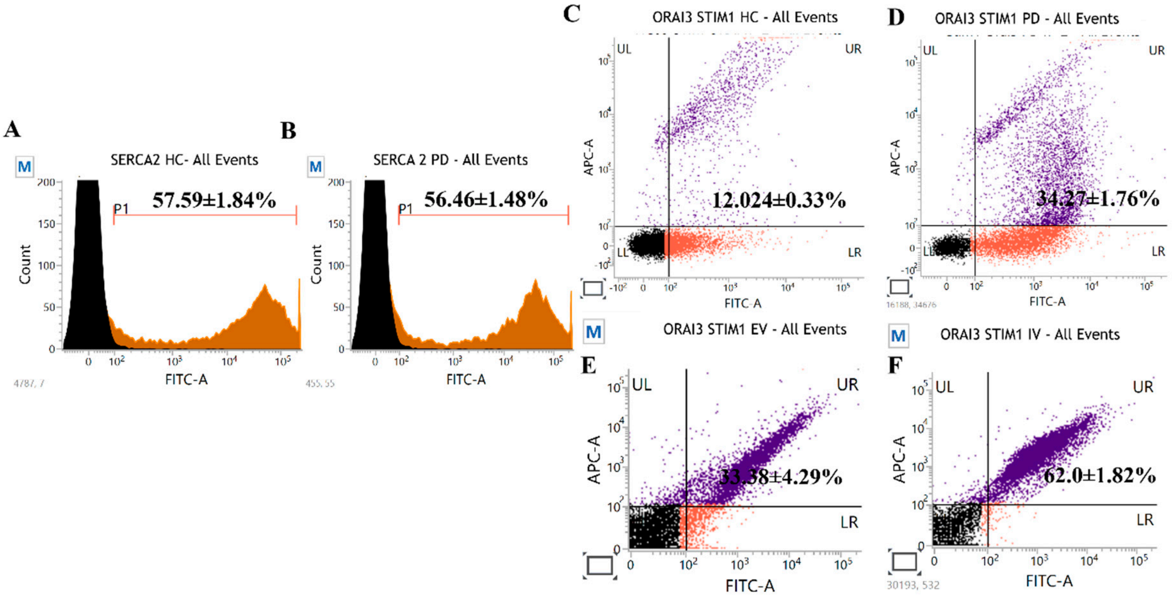

Figure S7. (A-B) Representative flow cytometry histogram of SERCA2 in HC **(A)** and PD **(B)** astrocytes. **(C-D)** Representative Scatter Plot of dual positive ORAI3 and STIM1 population of cells in HC **(C)** and PD **(D)** astrocytes. **(E-F)** Representative Scatter Plot of dual positive ORAI3 and STIM1 population of cells in EV **(E)** and IV **(F)** transfected U87 cells.

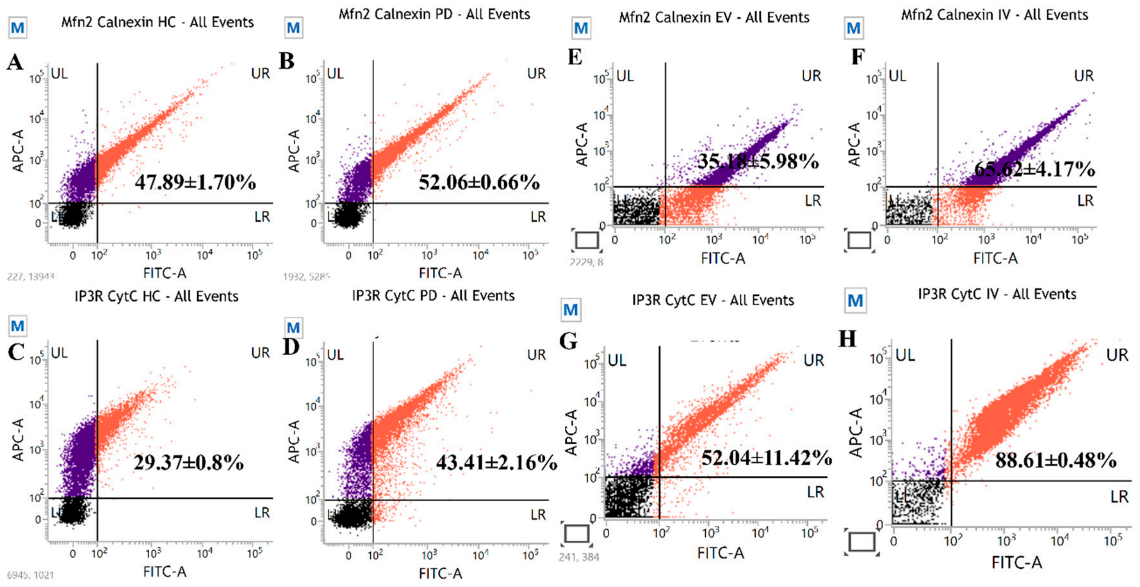

Figure S8. (A-B) Representative Scatter Plot of dual positive MFN2 and Calnexin population of cells in HC **(A)** and PD **(B)** astrocytes. (**C-D)** Representative Scatter Plot of dual positive

IP3R and Cytochrome C population of cells in HC **(C)** and PD **(D)** astrocytes. **(E-F)** Representative Scatter Plot of dual positive MFN2 and Calnexin population of cells in EV **(E)** and IV **(F)** transfected U87 cells. **(G-H)** Representative Scatter Plot of dual positive IP3R and Cytochrome C population of cells in EV **(G)** and IV **(H)** transfected U87 cells.

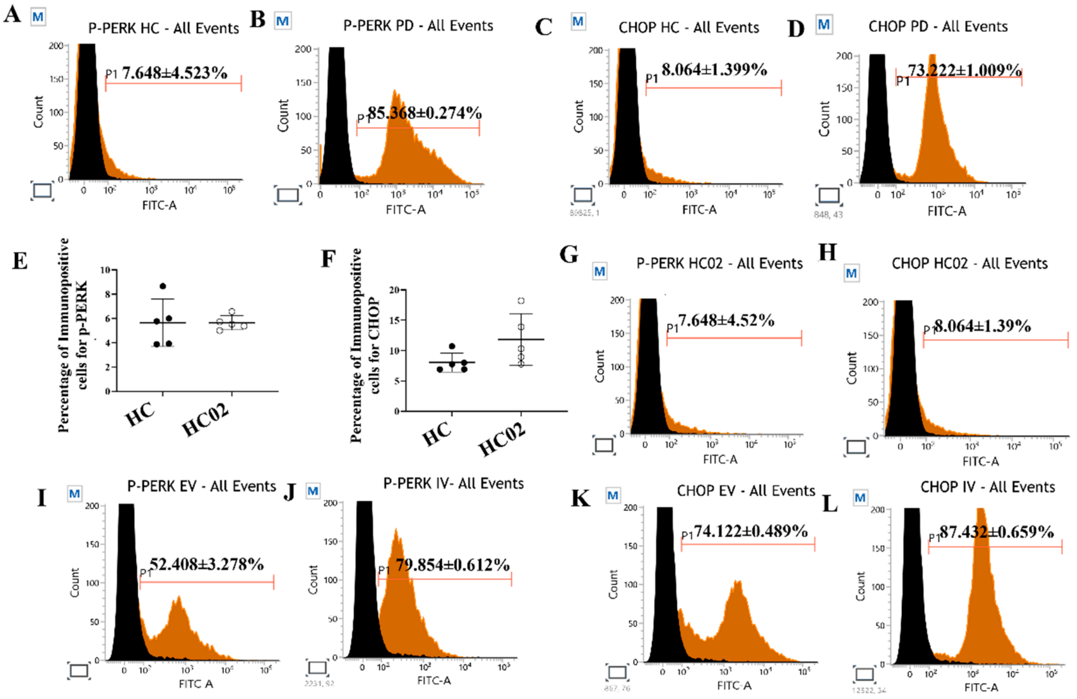

Figure S9. Flow Cytometry Histograms. (A-B) Representative flow cytometry histogram of p-PERK in HC **(A)** and PD **(B)** astrocytes. **(C-D)** Representative flow cytometry histogram of CHOP in HC **(C)** and PD **(D)** astrocytes. **(E)** Graphical representation of the flow cytometry quantified positive population for p-PERK in HC and HC02 astrocytes. (n=5) **(F)** Graphical representation of the flow cytometry quantified positive population for CHOP in HC and HC02 astrocytes. (n=5). **(G)** Representative flow cytometry histogram of p-PERK in HC02 astrocytes. **(H)** Representative flow cytometry histogram of CHOP in HC02 astrocytes. **(I-J)** Representative flow cytometry histogram of p-PERK in EV **(I)** and IV **(J)** transfected U87 cells. **(K-L)** Representative flow cytometry histogram of CHOP in EV **(K)** and IV **(L)** transfected U87 cells.
